# Multiple drivers of large‐scale lichen decline in boreal forest canopies

**DOI:** 10.1111/gcb.16128

**Published:** 2022-03-08

**Authors:** Per‐Anders Esseen, Magnus Ekström, Anton Grafström, Bengt Gunnar Jonsson, Kristin Palmqvist, Bertil Westerlund, Göran Ståhl

**Affiliations:** ^1^ Department of Ecology and Environmental Science Umeå University Umeå Sweden; ^2^ Department of Statistics, USBE Umeå University Umeå Sweden; ^3^ Department of Forest Resource Management Swedish University of Agricultural Sciences Umeå Sweden; ^4^ Department of Natural Sciences Mid Sweden University Sundsvall Sweden; ^5^ Department of Fish, Wildlife and Environmental Sciences Swedish University of Agricultural Sciences Umeå Sweden

**Keywords:** climate change, colonization, epiphytic lichens, extinction, forestry, long‐term monitoring, microclimate, nitrogen deposition

## Abstract

Thin, hair‐like lichens (*Alectoria*, *Bryoria*, *Usnea*) form conspicuous epiphyte communities across the boreal biome. These poikilohydric organisms provide important ecosystem functions and are useful indicators of global change. We analyse how environmental drivers influence changes in occurrence and length of these lichens on Norway spruce (*Picea abies*) over 10 years in managed forests in Sweden using data from >6000 trees. *Alectoria* and *Usnea* showed strong declines in southern‐central regions, whereas *Bryoria* declined in northern regions. Overall, relative loss rates across the country ranged from 1.7% per year in *Alectoria* to 0.5% in *Bryoria*. These losses contrasted with increased length of *Bryoria* and *Usnea* in some regions. Occurrence trajectories (extinction, colonization, presence, absence) on remeasured trees correlated best with temperature, rain, nitrogen deposition, and stand age in multinomial logistic regression models. Our analysis strongly suggests that industrial forestry, in combination with nitrogen, is the main driver of lichen declines. Logging of forests with long continuity of tree cover, short rotation cycles, substrate limitation and low light in dense forests are harmful for lichens. Nitrogen deposition has decreased but is apparently still sufficiently high to prevent recovery. Warming correlated with occurrence trajectories of *Alectoria* and *Bryoria*, likely by altering hydration regimes and increasing respiration during autumn/winter. The large‐scale lichen decline on an important host has cascading effects on biodiversity and function of boreal forest canopies. Forest management must apply a broad spectrum of methods, including uneven‐aged continuous cover forestry and retention of large patches, to secure the ecosystem functions of these important canopy components under future climates. Our findings highlight interactions among drivers of lichen decline (forestry, nitrogen, climate), functional traits (dispersal, lichen colour, sensitivity to nitrogen, water storage), and population processes (extinction/colonization).

## Graphical abstract

Thin, hair‐like lichens have important ecosystem functions in boreal forest canopies. We analyse how environmental drivers influence changes in occurrence and length of these lichens in the lower canopy of Norway spruce (*Picea abies*) over 10 years in managed forests in Sweden using data from >6000 trees in the National Forest Inventory. *Alectoria* and *Usnea* showed strong declines in southern‐central regions, whereas *Bryoria* declined in northern regions. Our analysis strongly suggests that the decline was mainly driven by industrial forestry, in combination with nitrogen deposition. Our study highlights interactions among drivers of lichen decline, functional traits and population processes.

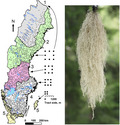

## 1 INTRODUCTION

The circumboreal forest constitutes 27% of global forest cover (Hansen et al., 2010). It is characterised by short growing seasons and cold winters with several months of snow cover, to which organisms have evolved various adaptations (Boonstra et al., 2016). Both natural (fire, wind, insects) and human‐induced disturbances (forestry, other land use) shape the structure, dynamics and function of the boreal forest, with about two‐thirds under some type of management, mainly for industrial wood production (Gauthier et al., 2015). These forests are also under strong pressure from environmental hazards, including habitat fragmentation, air pollution and climate change, with severe consequences for biodiversity and ecosystem services (Esseen et al., 1997; Moen et al., 2014; Pohjanmies et al., 2017). Global warming is stronger at the high latitudes of the boreal biome than in southern biomes, implying multiple risks to forest organisms (IPCC, 2013; Venäläinen et al., 2020).

Lichens, associations between fungi (mycobionts) and algae or cyanobacteria (photobionts), occur in forests worldwide and serve important ecosystem functions (Asplund & Wardle, 2017; Coxson & Howe, 2017; Porada et al., 2014, 2018). These are poikilohydric organisms, with metabolic activity that is dependent on water supplied from rain, dew or humid air and further influenced by species‐specific functional traits (Ellis, 2012; Ellis et al., 2021; Gauslaa, 2014; Green et al., 2011). Lichens are also well known for their sensitivity to air pollution such as sulphur (S), nitrogen (N) and heavy metals (Allen et al., 2019; Carter et al., 2017; Conti, & Cecchetti, 2001; Johansson et al., 2010; van Herk et al., 2003). Forestry has also caused widespread loss of lichens in boreal (Esseen et al., 2016; Hauck, 2011), temperate (Hauck et al., 2013; Nascimbene et al., 2013) and tropical forests (Benitéz et al., 2019). Given this list of vulnerabilities, lichens are excellent bioindicators of climate change in polar, alpine and temperate regions (Colesie et al., 2018; Ellis, 2013, 2019; Sancho et al., 2019), yet their responses in boreal areas are less known (Esseen et al., 2016; Hauck, 2009; Nascimbene et al., 2019).

Lichens are common throughout the boreal biome and form rather homogeneous communities on the forest floor and in canopies (Ahti, 1977; Payette & Delwaide, 2018). This study focuses on hair lichens within the filamentous genera *Alectoria*, *Bryoria* and *Usnea*, which are dominant epiphytic species in boreal forests (Esseen et al., 2015). These lichens show a clear vertical zonation, with dark *Bryoria* in the upper canopy and pale *Alectoria* and *Usnea* in the lower canopy (Coxson & Coyle, 2003). This zonation is mainly shaped by their colour and the function of species‐specific sun‐screening fungal pigments in the cortex (Färber et al., 2014). Niche differentiation is also linked to anatomical and morphological traits that influence uptake, storage and loss of water (Esseen et al., 2015, 2017; Gauslaa, 2014). Hair lichens dominate canopies in unmanaged boreal forests (Boudreault et al., 2015; Dettki, & Esseen, 1998). In fact, these lichens were so abundant that a century ago they were considered a serious problem for forestry, erroneously assumed to reduce tree growth, or even kill trees (Romell, 1922). Yet, these lichens do not significantly impair tree growth but instead provide important ecosystem functions, such as food for ungulates, particularly reindeer/caribou and small mammals, nesting material for birds, as well as microhabitat and food for invertebrates (Asplund & Wardle, 2017; Pettersson et al., 1995). Hair lichen abundance is also an easily visible indicator of general diversity of epiphytic lichenized and non‐lichenized fungi. These lichens are particularly sensitive to air pollution (Geiser et al., 2019, 2021) and forestry (Esseen et al., 1996, 2016; Hauck, 2011; Lesica et al., 1991; Stevenson & Coxson, 2007). Overall, however, we lack information about the speed and scale of changes in hair lichen populations and how different global change drivers interact and influence species with different functional traits.

Here, we have analysed how global change drivers influence changes in occurrence and length of hair lichens over 10 years in the lower canopy of Norway spruce (*Picea abies* (L.) Karst.; henceforth referred to as *Picea*) in managed forests in Sweden. Our analysis is based on >6000 trees surveyed 1993–2012 in the National Forest Inventory (NFI). We focus on changes in habitat quality by monitoring lichens when the host is present. This allowed us to examine how global change drivers interact and affect extinction and colonization processes. We hypothesized the following: (1) that lichen occurrence declines due to industrial forestry, driven by short logging cycles and an unfavourable microclimate (i.e. low light) in dense forests (Esseen et al., 2016); (2) that *Alectoria*, being associated with old forests (Esseen et al., 1996), declines faster than *Bryoria* and *Usnea*; (3) that lichen occurrence and thallus length (the length of the vegetative body) increase and recover in southern‐central regions in response to reduced anthropogenic N deposition since 1980 (Engardt et al., 2017). *Alectoria* and *Bryoria* are more sensitive to N deposition than *Usnea* (McCune & Geiser, 2009) and should respond faster to N reductions; (4) that climate change has genus‐specific effects on lichen occurrence. Here, a warmer and wetter climate, with more rain and less snow, should favour the occurrence of *Alectoria* and *Usnea*, as rainfall drives growth in these lichens (Phinney et al., 2021). In contrast, *Bryoria*, adapted to cold and dry climates with low rainfall (Esseen et al., 2017), should decrease in such climates.

## 2 MATERIALS AND METHODS

### 2.1 Study area

**FIGURE 1:**
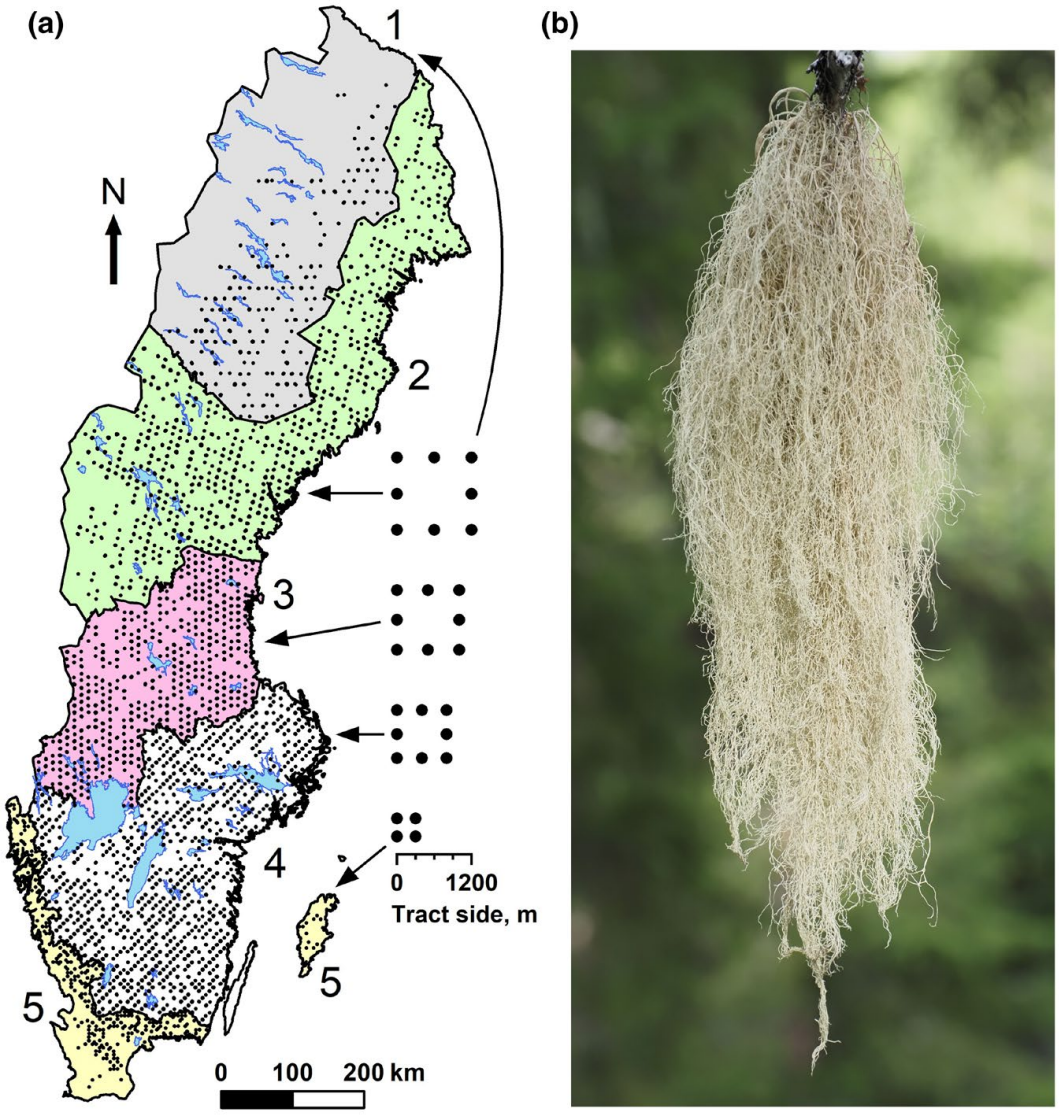
(a) Map of Sweden with five regions and National Forest Inventory plots clustered in tracts, with ≤4 (region 5) or ≤8 plots per tract (region 1–4). Region 1, northern boreal; 2, middle‐northern boreal; 3, southern‐middle boreal; 4, mainly hemiboreal; 5, temperate. (b) *Alectoria sarmentosa* (Witch's hair), a pale hair lichen associated with old coniferous forests in the boreal biome

The study area covers the whole of Sweden and spans latitudes 55–69°N (c. 1500 km). Sweden has 279,000 km^2^ of forest, of which 235,000 km^2^ is productive (site productivity ≥1 m^3^ ha^−1^ year^−1^; SLU, 2021). Ninety‐four percent of the productive forest is managed while 5.7% is formally protected (SCB, 2020). A large proportion of the protected forest forms intact landscapes along the eastern slopes of the Scandinavian Mountain range (Svensson et al., 2020). The study area covers large gradients in climate, vegetation, and anthropogenic impact. The climate ranges from cold temperate moist with snow in the north to warm temperate moist in the south, with warm summers in most of the country. The boreal zone covers most of the study area (Figure 1a) and is dominated by coniferous trees (*Picea*, *Pinus sylvestris* L.). The hemiboreal zone, a transition between the boreal and temperate zone, covers most of southern Sweden. The temperate zone forms a narrow belt in the south and southwest. It has broad‐leaved, deciduous trees (*Betula* spp., *Fagus sylvatica* L., *Acer* spp., *Fraxinus excelsior L*., *Quercus* spp., *Tilia cordata* Mill.) but also host conifers.

The current land use in Sweden is dominated by forestry (58% of the land area), followed by agriculture (8%; SLU, 2021). Reindeer husbandry is practiced in ~50% of the country and overlaps with forestry, which results in conflict of interest between traditional indigenous land use and modern industrial forestry (Horstkotte & Moen, 2019). Commercial forestry on a larger scale started with some selective cutting of large trees in the south‐central parts in the 18th century and harvest of trees for charcoal in the region of Bergslagen, central Sweden, followed by a northward expanding timber frontier during the 19th century (Östlund & Norstedt, 2021; Östlund et al., 1997). The high‐grading of large timber trees in old‐growth and multi‐layered forests was followed by a period of uneven‐aged forestry and was then gradually replaced by even‐aged forestry at around the mid‐20th century (Lundmark et al., 2013). Current forestry is dominated by clear‐cutting followed by planting of conifer monocultures, which are regularly thinned. Rotation cycles range from c. 50 years in the south to c. 120 years in the north (Fries et al., 2015). Eighty‐two percent of the productive forest is ≤100 years old (SLU, 2021). *Picea* dominates by volume (41%), followed by *P. sylvestris* (39%) and *Betula* spp. (12%). The growing stock has increased with 106% since the 1920s, at which time the growing stock had decreased during the late 19th century. This trend mainly results from the efficient, production‐oriented forestry, including cutting of unproductive stands, ditching, thinning, N fertilization and planting of seedlings on clear‐cuts (Östlund et al., 1997). Global warming, N deposition and elevated CO_2_ concentration have probably also enhanced forest growth (Elfving & Tegnhammar, 1996; Venäläinen et al., 2020).

The southern and central regions have earlier been exposed to high levels of SO_2_ pollution, which has substantially decreased since 1970 (Vestreng et al., 2007). N deposition has currently greater impact on forests and exceeds 10 kg N ha^−1^ year^−1^ in southern regions (Pihl Karlsson et al., 2011), while deposition is low in northern regions.

### 2.2 The Swedish NFI

We extracted data from the Swedish NFI, which is a broad‐scale monitoring program that provides information about wood resources, ecosystem services, biodiversity, and carbon sequestration (Fridman et al., 2014). The design includes stratification into five regions, with different sampling intensities and clustering of sample plots in square‐formed tracts (4–8 plots per tract; Figure 1a). The plots are circular with a radius of 10 m (314 m^2^) and are located around the tract perimeter (the length of tract side varies from 300 to 1200 m among regions). About 200 forest, vegetation and site variables are recorded on each plot. The NFI has a systematic program for quality assurance, including training, calibration, and control inventory (Fridman et al., 2014). The data in this study were collected by ~150 field personnel.

### 2.3 Lichen data

Hair lichens are surveyed by the NFI in permanent plots that are measured with a 10‐year interval. We used NFI data from two full inventory periods (IP; 1993–2002, IP1; 2003–2012, IP2) from plots in productive forest land (see above). Formally protected forests were excluded as they were not measured in IP1. Hence, our sample consists of managed forests with active forestry, but includes a small proportion of voluntarily set aside areas (~7%; SCB, 2020). We studied lichens on live *Picea* with DBH ≥150 mm and included all NFI plots with at least one such tree present. The sample tree was the first *Picea* encountered along the measurement direction in the plot. Therefore, a new tree could be selected in IP2, due to ingrowth, even if the old tree remained. The year and cause of death were recorded for trees that died or were cut. In such cases, a new tree was selected in IP2, while plots without sample trees were excluded. New plots were also included in IP2 when at least one *Picea* reached a DBH ≥150 mm. The sample included a total of 6140 trees (Table S1).

Occurrence and maximum thallus length (a proxy of lichen mass; Esseen, 2006; McCune, 1990) of *Alectoria*, *Bryoria* spp. and *Usnea* spp. were recorded on branches and the stem up to 5 m above ground. Our study is restricted to genus level as species are difficult to identify. However, *Alectoria* is only represented by *A. sarmentosa* (Ach.) Ach. (Figure 1b), a red‐listed (near‐threatened) species associated with old coniferous forests (Esseen et al., 1996). *Bryoria* is dominated by *B. capillaris* (Ach.) Brodo & D. Hawksw. (southern tendency) and *B. fuscescens* (Gyeln.) Brodo & D. Hawksw. (northern tendency), while *Usnea* is dominated by *U. dasopoga* (Ach.) Nyl. (widespread), followed by *U. subfloridana* Stirt. (southern tendency; Thell & Moberg, 2011). The lichens are henceforth referred to by their genus names.

### 2.4 Explanatory variables

We selected eight variables of ecological importance for the studied lichens (Table 1). Four were measured in NFI plots: diameter at breast height (DBH) and crown limit (CRL) for each tree, whereas basal area (BAS) and stand age (AGE) were measured at plot level. We also extracted a landscape context variable (MAT, mature forest), defined as the percent of forest ≥60 years old in a 100 m buffer around the centre of the plot. By using arcgis version 10.3, we extracted MAT from SLU Forest map (SLU, 2018), where forest age was estimated in 25 × 25 m cells by combining satellite and NFI data (Reese et al., 2003). MAT represents only 1 year (2005) as there were gaps in older data.

**TABLE 1:** Explanatory variables used in this study: scale of observations, time period and definition

Variable, unit, abbreviation	Scale	Time period	Definition
DBH, mm, DBH	Sample tree	1993–2002, 2003–2012	Diameter at breast height (1.3 m)
Crown limit, m, CRL	Sample tree	1993–2002, 2003–2012	Height of the lowest live branch
Basal area, m^2^ ha^−1^, BAS	10‐m radius plot	1993–2002, 2003–2012	Cross‐sectional area of live trees at breast height (1.3 m)
Stand age, year, AGE	10‐m radius plot	1993–2002, 2003–2012	Stand age weighed by basal area
Mature forest, %, MAT	100‐m radius	2005	Percent forest ≥60 years within 100 m from plot centre
Temperature, °C, TEMP	4 × 4 km	1993–2002, 2003–2012	Mean annual temperature, 10‐year mean
Rain, mm year^−1^, RAIN	4 × 4 km	1993–2002, 2003–2012	Annual precipitation for days with TEMP ≥0°C, 10‐year mean
N deposition, kg N ha^−1^ year^−1^, NDEP	20 × 20 km	1998–2002, 2003–2012	Annual N deposition, 5‐ and 10‐year mean

Gridded climate data (4 × 4 km) were obtained from the Swedish Meteorological and Hydrological Institute (SMHI, 2006; https://www.smhi.se/en/climate). We calculated mean annual temperature (TEMP) and mean total rain per year (RAIN) for each IP in all NFI plots. RAIN was defined as the sum of precipitation in days with mean temperature ≥0°C, during which lichens can be active. We also extracted deposition of atmospheric inorganic N (dry plus wet deposition; NDEP) from gridded data (20 × 20 km) based on the Match model (Robertson et al., 1999; https://www.smhi.se/data/miljo/atmosfarskemi). Mean annual N deposition was calculated for 1998–2002 (no data available for 1993–1997) and 2003–2012. The variables are henceforth referred to by their abbreviations (Table 1), and by adding ‘1’ or ‘2’ for the IPs. The change over time was calculated as ‘difference variables’ (e.g. TEMP∆ = TEMP2 − TEMP1).

### 2.5 Data analysis

#### 2.5.1 Lichen occurrence and thallus length

We first estimated the total number of live *Picea* ≥150 mm in each region and IP under a two‐phase sampling design by taking stratum area, clustering in tracts, area of sample plots and design weights for the sample trees into account (Toet et al., 2007; Appendix S1). We then calculated the occurrence proportion of each lichen on the sample trees as the ratio between the estimated number of trees with presence of the lichen and the estimated total number of trees. The occurrence proportion was estimated for all region and IP combinations, across all regions, and separately for remeasured and new trees in IP2, together with corresponding 95% confidence intervals (CI). The estimates are approximately unbiased due to the large sample size. The mean thallus length of each lichen on occupied sample trees and 95% CI were estimated based on the same principles as described above. We evaluated changes in regions and in the whole country by calculating 95% CIs for the difference in occurrence and thallus length between IPs. The change is significant (*p* < .05) if the CI do not overlap zero.

#### 2.5.2 Explanatory variables in all plots

We calculated summary statistics for the explanatory variables (Table 1) across all NFI plots with sample trees in each IP to evaluate the magnitude and direction of changes over time in these variables. Means and 95% CIs were calculated for all region and IP combinations, and across all regions. However, MAT was only summarized by region as data represented 1 year (see above).

#### 2.5.3 Explanatory variables in plots with remeasured trees

The trees that were remeasured (~50%) allowed us to examine how changes in lichen occurrence correlate with changes in variables over time in the plots. A lichen is either present (P) or absent (A) on a sample tree in each IP, and thus there are four occurrence trajectories (outcomes): persistence (PP), absence (AA), colonization (AP, ‘gain’) and extinction (PA, ‘loss’). We used Chi‐square tests to examine the association between trajectories and regions for each lichen. Extinction and colonization rates were calculated following Yalcin and Leroux (2018). The extinction rate was calculated as the ratio of the number of trees where the lichen had disappeared in IP2 divided by the number of trees where it was present in IP1. The colonization rate represented the ratio of the number of colonized trees in IP2 divided by the number of trees where the lichen was absent in IP1. We also constructed maps to depict patterns of extinction and colonization. Some overestimations of colonization and extinction rates are likely, as short thalli may have been overlooked.

We calculated the correlation coefficient (*r*) between all variable pairs to identify potential associations among the variables. We then used multinomial logistic regression (Hosmer et al., 2013) to identify the variables that best correlated with the occurrence trajectories and to examine the relationships. In such ‘multi‐outcome’ models the dependent variable has more than two levels, in our case four levels. We fitted ‘single variable’ logistic regression models for each of the seven variables measured in IP1, the corresponding difference variables, and MAT, as well as ‘multiple variable’ models. A single variable model includes one explanatory variable, but it may appear in several transformations. For model‐building, we used a multivariable fractional polynomials approach (Hosmer et al., 2013), which finds non‐linear transformations if sufficiently supported by the data, and removes weakly influential covariates by backward elimination (Sauerbrei & Royston, 1999). For this purpose, we used the r package mfp (Ambler & Benner, 2015). Although this package does not support multinomial logistic models, the suggestion by Hosmer et al. (2013) is that an individualized fitting approach, where separate binary logistic models are fitted, is useful for finding suitable non‐linear transformations. Without taking the sampling design into account, we used this approach for finding preliminary main‐effects models. After this stage, the preliminary main‐effect models were refitted taking the complex sampling design of the NFI into account (Appendix S2; cf. Ekström et al., 2018), after which we considered possible interactions between the main effects. Any model selected was considered preliminary until we evaluated its fit. We used the Akaike information criterion for the final model selection. Model performance was evaluated by calculating McFadden's pseudo *R* ^2^ (Menard, 2000).

The multiple‐variable multinomial logistic models were interpreted with odds ratios. Consider a model with a single continuous explanatory variable *x* and a response variable *Y*. Then, if *j* is one of the occurrence trajectories, the probability that *Y* = *j* is assumed to depend on the value of *x* and is denoted by $\mathrm{Pr}\left(Y=j|x\right)$. Absence was used as our baseline trajectory and is labeled as *Y* = 0. The odds ratio of trajectory *Y* = *j* (e.g. extinction) versus *Y* = 0 for a one‐unit increase in the explanatory variable is then given by

$${\text{OR}}_{j}=\frac{\mathrm{Pr}\left(Y=j|x+1\right)/\mathrm{Pr}\left(Y=0|x+1\right)}{\mathrm{Pr}\left(Y=j|x\right)/\mathrm{Pr}\left(Y=0|x\right)},$$

where $\mathrm{Pr}\left(Y=j|x\right)/\mathrm{Pr}\left(Y=0|x\right)$ is the odds at *x* that the trajectory is *j*, given that it is either *j* or 0. If there are other explanatory variables than *x* present in the model, these are kept fixed when computing OR*_j_*. The odds ratio OR*_j_* is a measure of how much more likely or unlikely (in terms of odds) it is for occurrence trajectory *j* to be present among those trees with a one‐unit increment in an explanatory variable *x* as compared to those with no increment in this variable, while holding the other explanatory variables fixed. The odds ratio is significantly different from 1 when the 95% confidence band does not overlap 1. The analyses were done with r version 4.0.3 (R Core Team, 2020).

## 3 RESULTS

### 3.1 Host trees

The number of sampled *Picea* decreased with 6% over time (Table S1). Of the trees, 52.4% in IP1 were remeasured, 17.5% were cut, 1.8% died and 28.4% were only measured once. The proportion of cut trees increased from 8.8% in region 1 to 21.0% in region 4. Accordingly, the proportion of new trees increased along the same gradient. One fifth (20.8%) of the plots in IP2 were in forests subject to thinning.

### 3.2 Lichen occurrence

**FIGURE 2:**
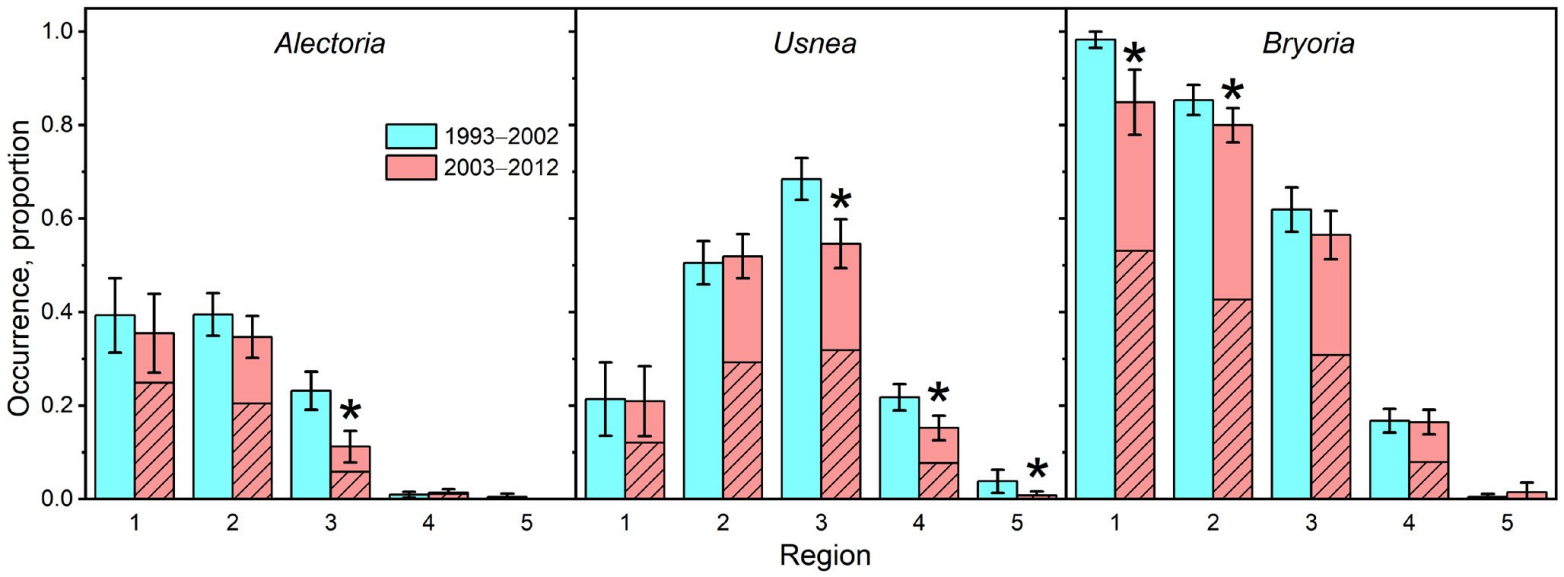
Estimated occurrence proportion (means and 95% CIs) of the studied lichens on *Picea* in two 10‐year periods by five regions in Sweden. The diagonal hatch pattern in the second period represents remeasured trees, while the upper, open column represents new trees. Stars indicate changes that are significant at *p* < .05

The occurrence proportion of all lichens decreased over time. *Alectoria* decreased from 0.205 ± 0.018 (mean ± error bounds for a 95% CI) to 0.173 ± 0.018 across the country, *Usnea* from 0.388 ± 0.020 to 0.342 ± 0.021 and *Bryoria* from 0.532 ± 0.016 to 0.506 ± 0.019 (Table S2). This corresponds to 1.70%, 1.25% and 0.50% relative loss rates per year, assuming constant loss rates over the 10‐year period. More than half of the *Alectoria* occurrences (51.6%) was lost in region 3 (Figure 2), followed by substantial decreases of *Usnea* in regions 3 (20.2%) and 4 (30.1%), whereas *Bryoria* decreased in regions 1 (13.6%) and 2 (6.3%). The geographic distribution of *Alectoria* was substantially reduced in region 3 (Figure S1). The distributions of *Bryoria* and *Usnea* were more stable.

**FIGURE 3:**
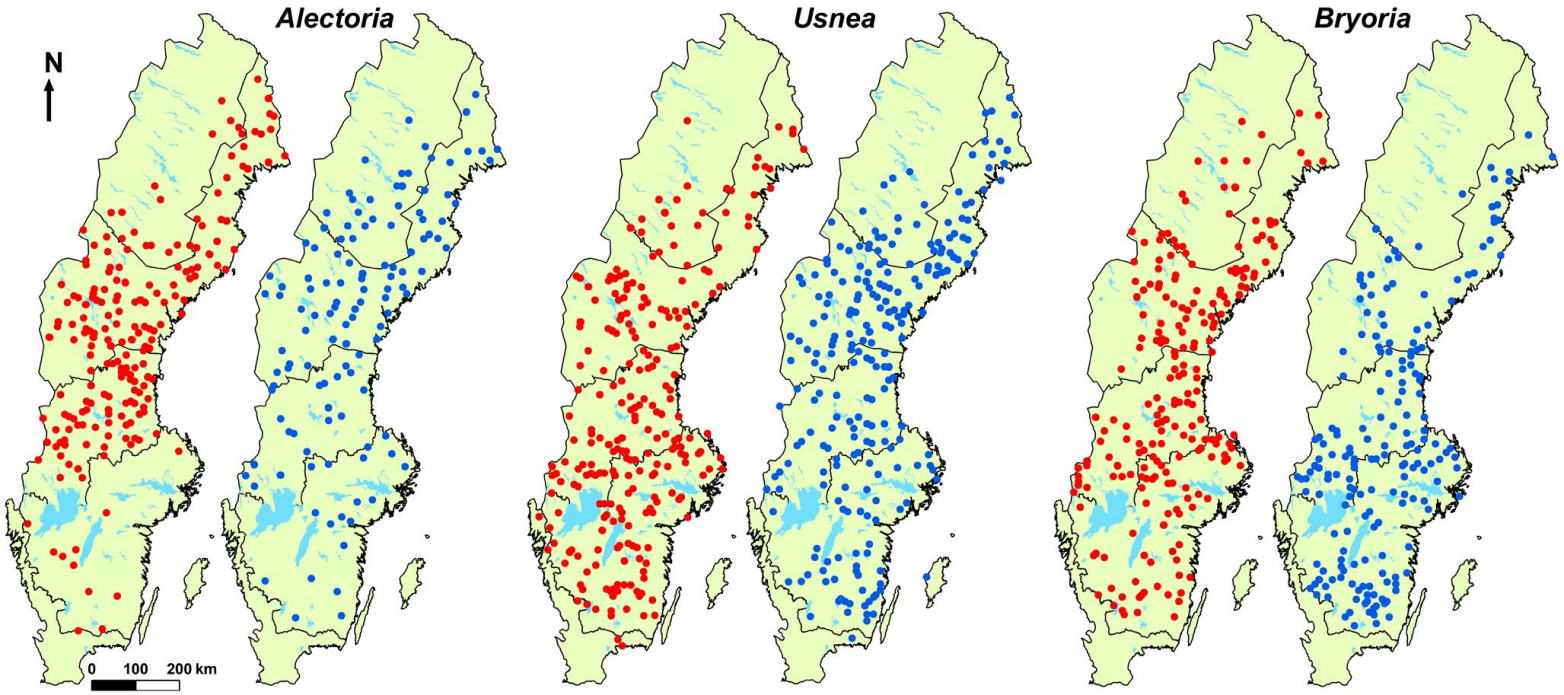
Distribution of extinction (filled red circles) and colonization (filled blue circles) of the studied lichens on remeasured *Picea* over a 10‐year period (1993–2002; 2003–2012). *N* = 2196

Remeasured trees had higher lichen occurrence than new trees in IP2, particularly in *Alectoria*, 0.218 versus 0.130, respectively (Table S2). Extinction and colonization rates were high in several regions (Table 2; Figure 3), indicating substantial turnover, especially in *Alectoria* and *Usnea*. The Chi‐square test of association between occurrence trajectory and region was significant (*p* < .05) for all lichens, showing that trajectories varied by region.

**TABLE 2:** Occurrence trajectories of studied lichens over 10‐year on remeasured trees included in multinomial logistic regression models. Values in parentheses represent extinction and colonization rates (see 2, 2.5.3 for explanation). *N* = 2196

Lichen	Region	Occurrence trajectory, no. of trees				Net change
		Persistence	Extinction	Colonization	Absence	
*Alectoria*	1	51	17 (0.25)	22 (0.21)	85	+5
	2	167	122 (0.42)	76 (0.23)	255	−46
	3	26	77 (0.75)	24 (0.07)	321	−53
	4	3	10 (0.77)	19 (0.02)	772	+9
	5	0	1 (1.00)	0 (0.00)	148	−1
	1–5	247	227 (0.48)	141 (0.08)	1581	−86
*Usnea*	1	18	17 (0.49)	20 (0.14)	120	+3
	2	216	78 (0.27)	122 (0.37)	204	+44
	3	243	69 (0.22)	50 (0.36)	87	−19
	4	110	96 (0.47)	58 (0.10)	540	−38
	5	1	6 (0.86)	2 (0.01)	140	−4
	1–5	587	266 (0.31)	252 (0.19)	1091	−14
*Bryoria*	1	152	20 (0.12)	3 (1.00)	0	−17
	2	449	90 (0.17)	54 (0.67)	27	−36
	3	216	67 (0.24)	68 (0.41)	97	+1
	4	83	72 (0.46)	82 (0.13)	567	+10
	5	0	1 (1.00)	0 (0.00)	148	−1
	1–5	900	250 (0.22)	207 (0.20)	839	−43

### 3.3 Thallus length

The length of *Alectoria* in IP1 (mean 18.1 cm across regions) was twice as high as *Usnea* (8.6 cm), with *Bryoria* intermediate (13.6 cm). Length of *Bryoria* increased slightly over time in regions 3–5 and *Usnea* in regions 2–4, whereas *Alectoria* did not change (Figure S2). However, the change in length across the country was only significant for *Usnea*.

### 3.4 Explanatory variables in all plots

**FIGURE 4:**
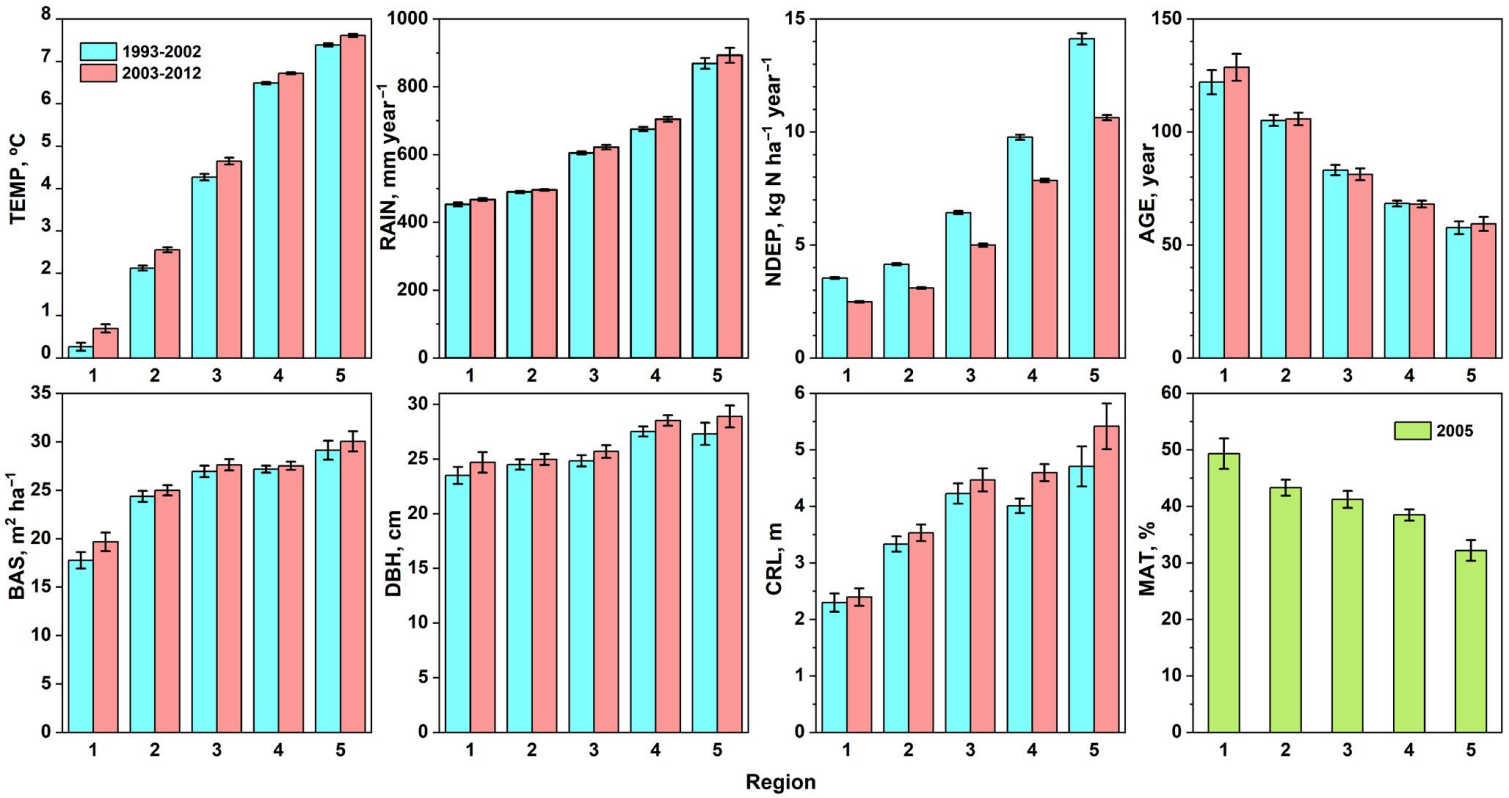
Summary of explanatory variables (means and 95% CIs) in two 10‐year periods by five regions, except for MAT, which is based on data from 2005. BAS, basal area; CRL, crown limit; DBH, diameter at breast height; MAT, mature forest

The individual NFI plots spanned a large range in TEMP (−1.7 to 8.6°C) and RAIN (335–1290 mm). All variables showed clear latitudinal trends from north (region 1) to south (region 5; Figure 4). The warming over time (TEMP∆) was strongest (0.4°) in northern regions, while RAIN∆ increased with only 13 mm. NDEP1 was four times higher in region 5 than in region 1, with a reduction over time in all regions. AGE1 increased twofold from region 5 to 1 but was rather stable over time, due to plot turnover. AGE2 was 21 years lower in new plots than in remeasured plots (means of 72 and 93 years, respectively). BAS, DBH and CRL increased towards southern regions. Over time, BAS increased with 0.6 m^2^ ha^−1^ and DBH with 0.9 cm across regions. CRL increased with 0.4 m, with the largest increase in regions 4 and 5 (0.6–0.7 m). MAT decreased towards the south reflecting higher degree of forest fragmentation.

### 3.5 Explanatory variables in plots with remeasured trees

**FIGURE 5:**
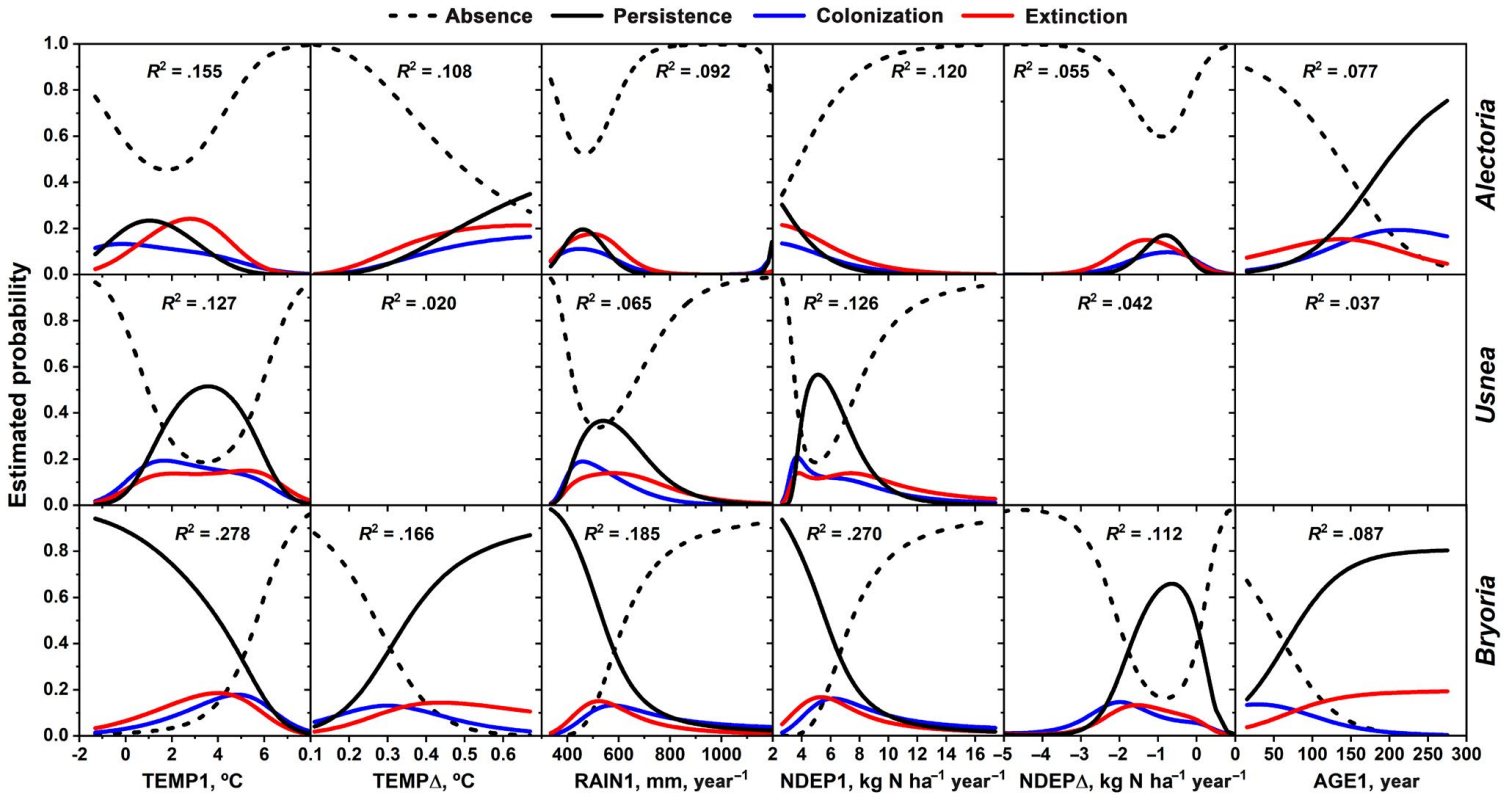
Estimated probability of occurrence trajectories for the studied lichens on remeasured *Picea* over 10 years for six explanatory variables based on single variable multinomial logistic regression models. Only models with *R* ^2^ > .05 are shown. Variable transformations and *p*‐values are found in Table S4. *N* = 2196

The correlations were relatively weak between most variable pairs, but significant due to the large sample size (Table S3). However, there was a strong relationship between NDEP1 and RAIN1 (*r* = .910). The single‐variable logistic models had highly significant (*p* < .001) slope coefficients for most variable transformations in the occurrence trajectories (Table S4). The trajectories correlated best with TEMP1 and NDEP1 in all lichens (Figure 5). TEMP∆ and AGE1 had higher *R* ^2^ for *Alectoria* and *Bryoria* than for *Usnea*. The overall pattern of the trajectories for RAIN1 and NDEP1 were similar, with highest *R* ^2^ for *Bryoria*. BAS, DBH, CRL, their corresponding difference variables, and MAT had *R* ^2^ < .05 (Table S4). The difference between extinction and colonization probabilities in Figure 5 indicates the direction and magnitude of changes in lichen occurrence across the range of each variable.

**FIGURE 6:**
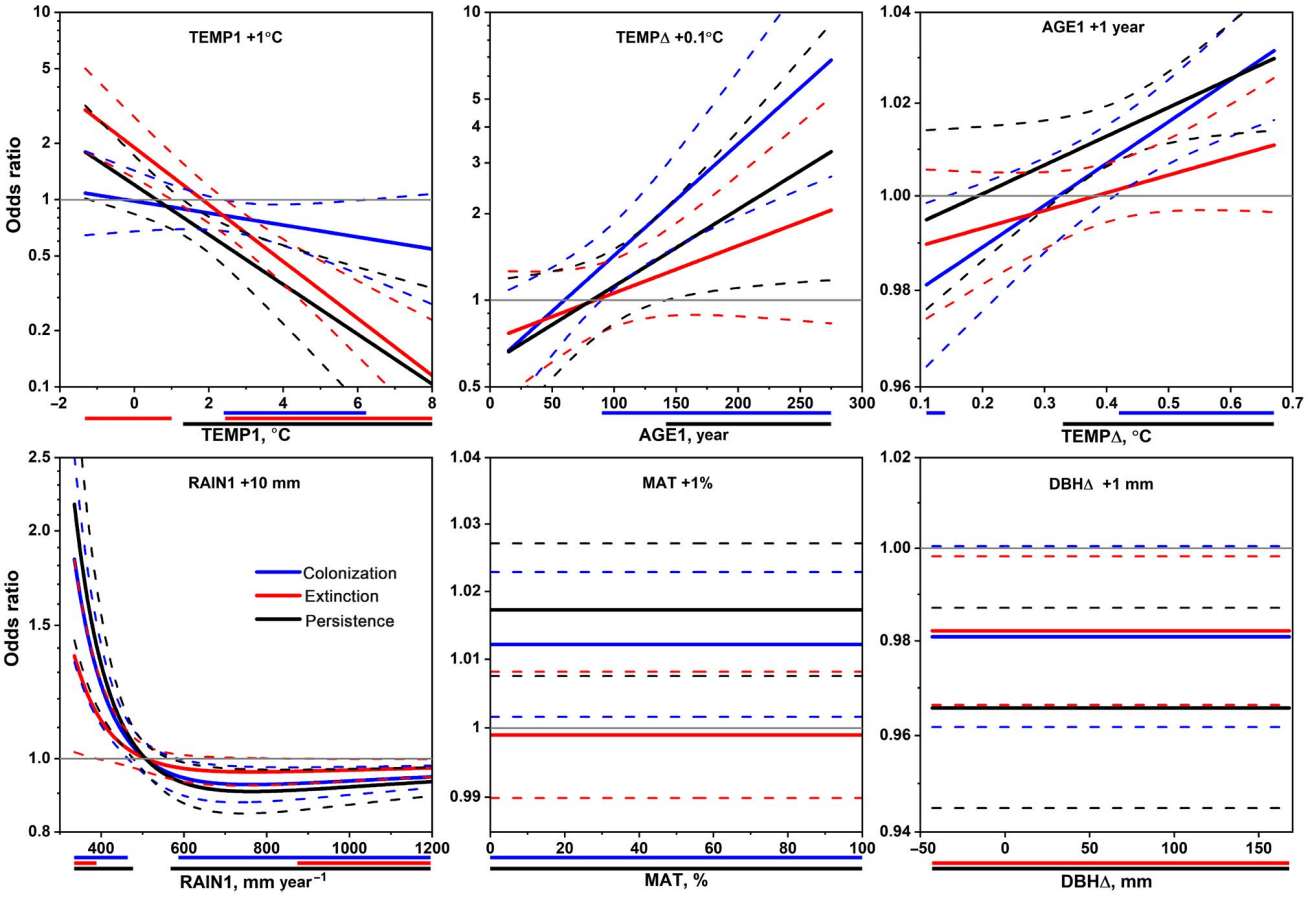
Odds ratio functions (solid lines, log‐scale) for occurrence trajectories of *Alectoria* over 10 years on remeasured *Picea* with 95% confidence bands (dashed lines) when adding one unit to either TEMP1, TEMP∆, AGE1, RAIN1, MAT or DBH∆ while holding other variables constant in multiple variable multinomial logistic regression models. Note that AGE1 interacts with TEMP∆ and is displayed on the *x*‐axis, while TEMP∆ is displayed on the *x*‐axis for AGE1. Thin grey horizontal lines indicate an odds ratio of 1.0. Horizontal coloured lines below the *x*‐axis indicate the intervals where the odds ratios of the trajectories are significantly different from 1.0. DBH, diameter at breast height; MAT, mature forest

**FIGURE 7:**
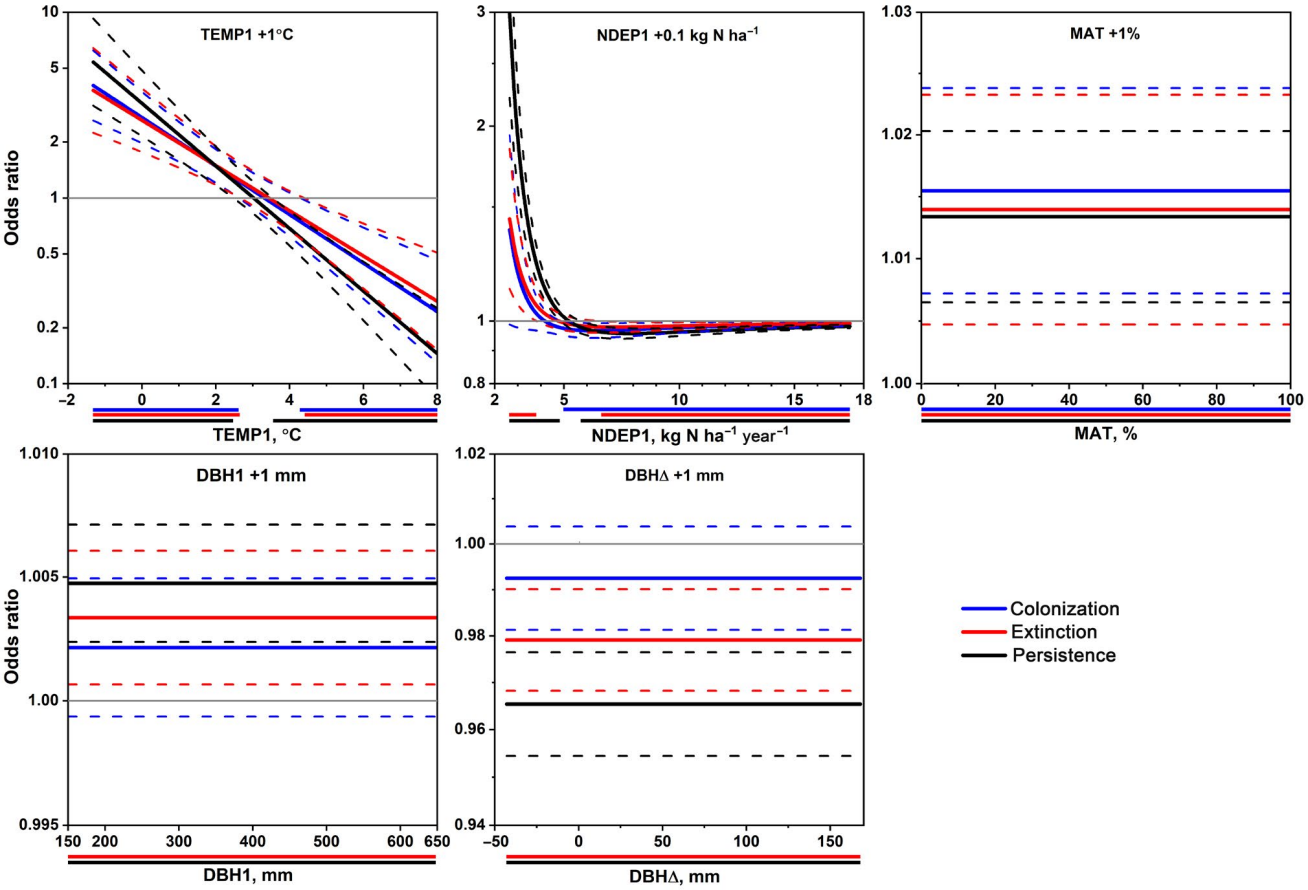
Odds ratio functions (solid lines, log‐scale) for occurrence trajectories of *Usnea* over 10 years on remeasured *Picea* with 95% confidence bands (dashed lines) when adding one unit to either TEMP1, NDEP1, MAT, DBH1 or DBH∆ while holding other variables constant in multiple variable multinomial logistic regression models. See Figure 6 for additional explanations. DBH, diameter at breast height; MAT, mature forest

**FIGURE 8:**
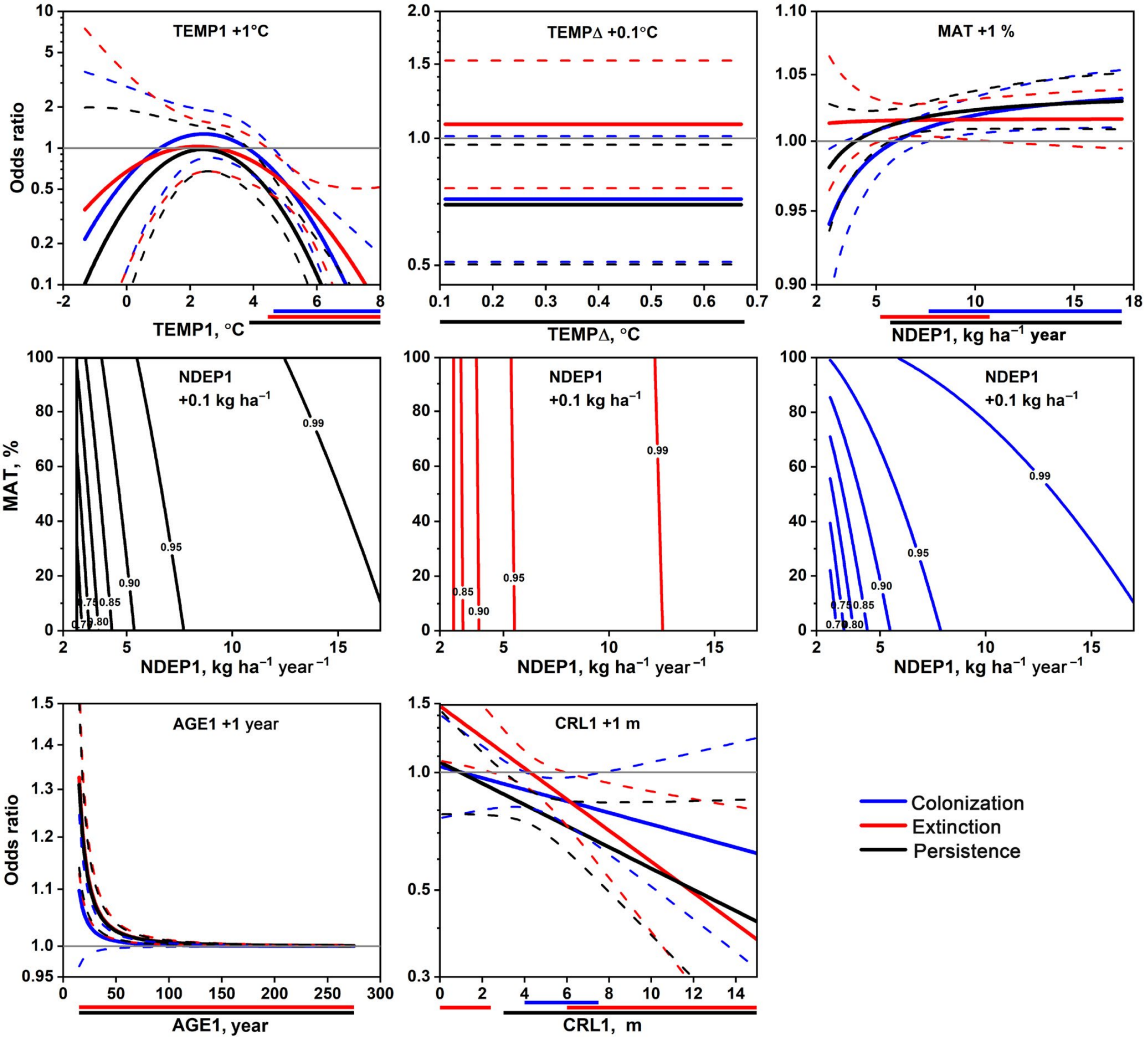
Odds ratio functions (solid lines, log‐scale) for occurrence trajectories of *Bryoria* over 10 years on remeasured *Picea* with 95% confidence bands (dashed lines) when adding one unit to either TEMP1, TEMP∆, MAT, NDEP1, AGE1 or CRL1 while holding other variables constant in multiple variable multinomial logistic regression models. Note that MAT interacts with NDEP1, which is why NDEP1 is displayed on the *x*‐axis for odds ratios of MAT, whereas odds ratios of NDEP1 are displayed as contour plots with MAT on the *y*‐axis. For NDEP1, all odds ratios are significant for persistence and extinction, while odds ratios for colonization are significant where MAT is below 74%. See Figure 6 for additional explanations. CRL, crown limit; DBH, diameter at breast height; MAT, mature forest

The multiple‐variable model for *Bryoria* had higher explanatory power (*R* ^2^ = .337) than models for *Alectoria* (*R* ^2^ = .218) and *Usnea* (*R* ^2^ = .186; Table S5). The models included five or six variables, but only two of these were difference variables (TEMP∆ and DBH∆). Figures 6, 7, 8 show the odds ratios for colonization, extinction and persistence relative to absence when a variable is increased while holding other variables fixed. Adding 1°C to TEMP1 in the *Alectoria* model increased the odds of extinction in colder climates whereas these odds, and those of persistence, decreased in warmer climates (Figure 6). Odds of colonization decreased slightly at intermediate climates. The same addition for *Usnea* increased the odds of all trajectories in colder climates, whereas these odds decreased in warmer climates in both *Usnea* (Figure 7) and *Bryoria* (Figure 8). Adding 0.1°C to TEMP∆ markedly increased odds of colonization and persistence for *Alectoria* in older forests, due to interaction with AGE1, but did not affect extinction. The same change in *Bryoria* decreased the odds of persistence. An increase of RAIN1 with 10 mm in *Alectoria* increased the odds of all trajectories in drier climates but decreased these odds slightly in wet climates. RAIN1 was not significant in multiple‐variable models for other lichens. For *Usnea*, an increase of NDEP1 with 0.1 kg N ha^−1^ markedly increased the odds of extinction and persistence at low values but reduced odds of all trajectories at intermediate‐high values. The same increase for *Bryoria* reduced the odds of all trajectories, but the odds were affected by interaction between NDEP1 and MAT. Changes in forest variables had less impact on odds than changes in TEMP, RAIN and NDEP. However, MAT, with low *R* ^2^ as a single variable, was included in multiple variable models for all lichens. Adding 1% to MAT slightly increased the odds of colonization and persistence in *Alectoria*, all trajectories in *Usnea*, and colonization and persistence at intermediate‐high values of NDEP1, as well as extinction at intermediate values in *Bryoria*. An increment of CRL1 in *Bryoria* with 1 m slightly reduced odds of colonization at intermediate values, increased odds of extinction at low values but decreased these odds at high values, while odds of persistence decreased at CRL1 ≥3 m.

## 4 DISCUSSION

Habitat loss, fragmentation and degradation are main drivers behind loss of biodiversity in forests across the globe (Fischer & Lindenmayer, 2007; Haddad et al., 2015). Here, we show that hair lichens decline in managed forests across broad spatial scales even when an important host tree (*P. abies*) in the boreal biome is present. Our key finding of significant declines in Sweden over 10 years is alarming, as these lichens already experienced large‐scale declines also before the 1990s (Bruteig, 1993; Esseen et al., 2016; Kuusinen et al., 1990). The speed of the *Alectoria* decline in region 3 (~50% loss) is remarkable, as the distribution of this lichen had once been centred in this region of Fennoscandia (Ahlner, 1948). We base our interpretation of causal mechanisms on state and change in variables influencing lichen occurrence on all trees, and on logistic regression models for occurrence trajectories on remeasured trees, where each tree was monitored over time.

### 4.1 Forestry and forest structure

Our first hypothesis, predicting that industrial forestry causes a decline of hair lichens is supported by the following points: (1) Declines were observed in all regions, and forestry is the only driver with significant impact across the country. (2) The estimated annual loss rates of the lichens (0.5%–1.7%) are comparable to that ~1% of the forest area is logged each year, mainly by clearcutting of older forests. Moreover, the proportion of logged forests older than 120 years has increased since 2000 (Fries et al., 2015). Logging of old, multi‐layered forests with long continuity of cover (Svensson et al., 2019) is harmful for these lichens (Dettki & Esseen, 1998; Esseen et al., 1996). (3) The new trees in IP2 had fewer lichens than remeasured trees, showing that the transformation from naturally to artificially regenerated forests contributed to the decline. (4) Short rotation cycles are incompatible with accumulation of high standing crop of these lichens (Dettki & Esseen, 2003). (5) *Alectoria*, with the strongest association to old forests, showed the steepest decline, supporting both our first and second hypothesis. This is not explained by growth responses as *Alectoria* grows faster than *Bryoria* and *Usnea* in lower canopy (Phinney et al., 2021). Instead, dispersal limitation and low propagule availability contribute to the scarcity of *Alectoria* in production forests. This lichen disperses with large thallus fragments, which are less suitable for long‐distance dispersal (Dettki et al., 2000; Esseen et al., 1996), whereas *Bryoria* and *Usnea* disperse effectively by numerous small fragments and symbiotic propagules (Clerc, 2011; Esseen, 1985), which rapidly colonise tree saplings (Hyvärinen et al., 1999). (6) MAT was included in the multiple‐variable models for all lichens, suggesting that landscape fragmentation also influenced occurrence trajectories. The lower MAT in southern regions implies greater proximity to edges and increased risk for adverse effects on microclimate as well as higher N deposition (Harper et al., 2015; Schmidt et al., 2017). It also reflects less adjacent forests functioning as source of dispersal propagules (Bartemucci et al., 2022; Dettki et al., 2000).

The low explanatory power of forest variables is partly due to the overriding effects of the large latitudinal gradients in climate and N deposition. However, it is well established that dense, productive conifer forests with high basal area provide poor growth conditions for lichens (Esseen et al., 2016; Hauck, 2011). Productive forests with a high CRL have few large, live branches in the lower canopy. Dead branches may be numerous but are smaller than live branches. Hence, the increase in CRL in all regions suggests that substrate limitation contributed to the decline. Moreover, lichen growth in lower canopy is substantially limited not only by low light in dense stands, such as spruce plantations, but also by less frequent hydration, due to rain interception in upper canopies. *Bryoria*, with dark cortical pigments, suffers more from low light conditions than *Alectoria* and *Usnea*, with pale pigments, as the former has a higher light compensation point for photosynthesis (Coxson & Coyle, 2003; Gauslaa et al., 2020). *Alectoria* and *Usnea* are adapted to the shaded lower canopy while *Bryoria* is adapted to the exposed upper canopy and open forests (Esseen et al., 2017; Färber et al., 2014). Successional changes as forests become older and darker cause an upward vertical displacement of *Bryoria* and may partly explain declines on remeasured trees.

### 4.2 Nitrogen

The decline on remeasured trees and similarly recorded declines in protected forests (Jönsson et al., 2017) show that other drivers also affected the lichens. Although thallus length increased slightly in some regions, we found no increase in occurrence in southern‐central regions in response to the reduced anthropogenic N deposition since ~1980 (Engardt et al., 2017). Moreover, the regression coefficients for NDEP∆ were not significant in the multiple variable models when other variables were included. Therefore, our third hypothesis predicting a recovery following the N reductions was not supported. Instead, we found strong evidence that high N deposition continues to limit occurrence of hair lichens. NDEP1 had significant explanatory power in the single variable models for all lichens as well as in the multiple variable models for *Bryoria* and *Usnea*. However, RAIN1 replaced NDEP1 in the multiple model for *Alectoria*. These variables were strongly correlated, and the odds ratio plots for RAIN1 in *Alectoria* and NDEP1 in *Usnea* were similar. The odds of extinction in *Alectoria* exceeded the odds of colonization at high rainfall (with high N), suggesting that N deposition contributed to its decline. High rainfall with a sufficient and not too high N concentration boosts growth of this lichen (Phinney et al., 2021).

High N deposition causes an imbalance between the photo‐ and mycobionts, loss of the integrity of the lichen symbiosis and increased risk for parasitic fungal attacks (Carter et al., 2017). The harmful effects of N also accumulate over time (Johansson et al., 2012), causing time lags in responses. Hair lichens are oligotrophic and N‐sensitive and have critical loads (above which N is harmful) in the range of 2–6 kg N ha^−1^ year^−1^ (Esseen et al., 2016; Geiser et al., 2019, 2021; McCune & Geiser, 2009). Our data, based on the MATCH model, show that N deposition exceeded 5 kg N ha^−1^ year^−1^ in most of the south‐central regions. However, N deposition in *Picea* forests can be up to 6 kg N ha^−1^ year^−1^ higher than estimates from this model (Karlsson et al., 2019). Hence, N deposition likely exceeded critical loads for all lichens in southern‐central regions. *Alectoria* is more sensitive to N than *Usnea* (Esseen et al., 2016; McCune & Geiser, 2009) accelerating its decline. N deposition contribute to the low occurrence of *Bryoria* in southern‐central regions but cannot explain its northern decline.

Weldon and Grandin (2021) found only weak recovery of epiphytic lichens over the last 20 years in southern Sweden. Our data suggest >30 year recovery lag of hair lichens following the improved air quality, in accordance with studies from central Europe showing strong recovery delays in response to lower S and N deposition (Hauck et al., 2013; Schmitz et al., 2019).

### 4.3 Climate

The occurrence trajectories correlated with TEMP1 in the models, reflecting the lichens macroclimate preferences along the latitudinal gradient (Esseen et al., 2016). More importantly, TEMP∆ was significant for *Alectoria* and *Bryoria* after controlling for other variables in multiple variable models. An increase of only 0.1° increased odds of colonization and persistence in *Alectoria* but decreased odds of persistence in *Bryoria*, whereas *Usnea* was not affected. Hence, our fourth hypothesis, predicting genus‐specific responses to climate change, was supported. Because *Bryoria* needs more light for positive carbon gain (see above), it likely suffers more from respiration losses in low‐light conditions during warmer autumns and winters than *Alectoria* and *Usnea*, contributing to its decline in dense forests and northern regions.

The small increase in rain had marginal effect on lichen occurrence. However, precipitation is predicted to increase with 10%–40% during this century, particularly in northern regions (Sjökvist et al., 2015). *Alectoria* and *Usnea* grows better in wetter and warmer climates and may expand northwards unless light and dispersal are limiting. *Alectoria* grows faster than *Usnea* across continental to oceanic macroclimates (Phinney et al., 2021) due to a larger internal water storage pool (Eriksson et al., 2018; Esseen et al., 2015), extending hydration and carbon gain. *Bryoria*, in contrast, has large external water storage pool (Esseen et al., 2017) causing suprasaturation depression of photosynthesis, and in situ decomposition if exposed to prolonged hydration (Coxson & Coyle, 2003). This lichen may face periodic dieback in future wetter climates (Gauslaa, 2014; Goward, 1998), shrinking its distribution, unless forests become more open.

Climate‐induced disturbances such as heatwaves, windstorms, snow/ice, wildfire and insect outbreaks also influence epiphytic lichens (Boudreault et al., 2009; Esseen, 1985, 2019; Malíček et al., 2019; Miller et al., 2018). A severe storm struck southern Sweden 2005 resulting in large‐scale windthrow, with subsequent outbreaks of spruce bark beetle (Valinger & Fridman, 2011), decreasing the number of potential host trees. Storms also damage hair lichens, as they are susceptible to fragmentation (Esseen & Renhorn, 1998). Overall, however, stochastic disturbances had less impact on lichens than anthropogenic stressors in the present study. Forest disturbances are predicted to increase in the boreal biome under future climates (Seidl et al., 2017; Venäläinen et al., 2020) and constitute potential threats to epiphytic lichens.

### 4.4 Implications

The low occurrence and short length of the lichens, compared with natural forests (Esseen, 2019), implies that managed boreal forests have substantially lower standing crop than natural forests. This is reinforced by that we studied NFI plots with larger trees, which usually support higher lichen mass than smaller trees (Esseen et al., 1996). Clear‐cuts and young, managed stands have none or low mass of these lichens (Dettki & Esseen, 1998). Loss of lichens has cascading effects on trophic interactions and ecosystem function across the boreal biome. Hair lichens constitute supplementary and emergency fodder for reindeer (and caribou) during migration and winter (Heggberget et al., 2002). A rich supply of *Bryoria* in the lower canopy or on top of snowpack may be critical for their survival when terricolous lichens (*Cladonia* spp.) are unavailable under thick and/or hard snow and ice. These lichens have historically been important for reindeer husbandry in Fennoscandia (Berg et al., 2011), but the value of this component has increased due to the concurrent decline of terricolous lichens (Horstkotte & Moen, 2019; Sandström et al., 2016). Loss of hair lichens will also affect other ungulates and small mammals and decrease abundance of canopy‐living insects and spiders, constituting important prey for birds (Pettersson et al., 1995).

Sweden has had programmes for conservation of biodiversity in managed forests since the 1980s, focusing on retention (since the 1990s) and forest certification (Gustafsson & Perhans, 2010; Simonsson et al., 2015). Although retention of live and dead trees through the regeneration phase can mitigate some harmful effects of forestry on biodiversity (Fedrowitz et al., 2014; Rosenvald & Lõhmus, 2008), our results suggest that the current application of retention forestry in even‐aged silviculture cannot support high standing crop of hair lichens. Instead, natural disturbance‐based management strategies applied at multiple scales have large potential to maintain heterogeneity, microclimates, processes and biodiversity in forest ecosystems (Kuuluvainen et al., 2021; Messier et al., 2019). However, it is a challenge to integrate such multi‐scale conservation approaches in the intensively managed production forest matrix (Felton et al., 2020). A spectrum of methods must be applied to secure substrates and suitable microclimates for hair lichens and other epiphytes under future climate scenarios (Ellis & Eaton, 2021), including: (1) uneven‐aged, continuous‐cover forestry, such as partial cutting (Stevenson & Coxson, 2007), (2) longer rotation cycles (Dettki & Esseen, 2003), (3) preservation of groups or large patches of lichen‐rich trees to reduce edge influence (Esseen, 2019; Stevenson & Coxson, 2003), (4) variable density thinning to diversify substrates and microclimates (Kuuluvainen et al., 2021; Lehmkuhl, 2004) and (5) spatial planning to protect old, lichen‐rich forests such that propagule availability is secured throughout landscapes (Dettki et al., 2000).

## 5 CONCLUSION

This is the first study of drivers and changes in dominant canopy lichens based on a large probability sample from a latitudinal gradient across the boreal biome. The rapid decline in only 10 years is a stark warning that hair lichens gradually lose their ecological functions in managed boreal forests, with cascading effects on trophic interactions and ecosystem function. Our analysis strongly suggests that industrial forestry, in combination with N deposition, is the main driver of this decline. *Alectoria* and *Usnea* decreased in southern‐central regions where forests are more productive, denser, have shorter logging cycles and are subjected to higher N deposition than northern regions, where only *Bryoria* decreased. Logging of forests with long continuity of tree cover and dispersal limitation contributed to the steep decline of the old‐growth‐associated *Alectoria*. Warming correlated with occurrence trajectories of *Alectoria* and *Bryoria*, emphasizing that poikilohydric canopy‐living organisms respond directly to changes in microclimate as driven by climate change (De Frenne et al., 2021; Smith et al., 2018). Forest management must apply a multitude of approaches to secure the ecosystem functions of these important canopy components under future climate scenarios. Our findings highlight interactions among drivers of lichen decline (forestry, N deposition, climate change), functional traits (lichen colour, dispersal, sensitivity to nitrogen, water storage) and population processes (extinction, colonization). Long‐term monitoring (this study) must be combined with experimental approaches to better understand how global change drivers influence hair lichens and other epiphytes across the boreal biome.

## CONFLICT OF INTEREST

The authors declare no conflict of interest.

## AUTHOR CONTRIBUTIONS

P‐AE designed the study and wrote the manuscript with help from all co‐authors. BW extracted the NFI data and P‐AE the other data, AG estimated lichen occurrence and length, ME performed the logistic regressions and P‐AE performed the other analyses. ME, AG, GS and BW contributed to statistical aspects, while BGJ and KP contributed to ecological aspects. All authors contributed critically to the drafts and gave final approval for publication.

## Supporting information

Fig S1‐S2

Table S1‐S5

Appendix S1

Appendix S2

## Data Availability

The data that support the findings of this study are openly available in Dryad at https://doi.org/10.5061/dryad.2ngf1vhq5.
