# Supplementary material for: Multiple drivers of large‐scale lichen decline in boreal forest canopies
Source: Glob Chang Biol. 2022 Mar 8;28(10):3293–309. doi: 10.1111/gcb.16128 (PMC9310866; doi:10.1111/gcb.16128)

**Supporting Information Figure S1.** Distribution of studied lichens on *Picea abies* in managed forests in Sweden in 1993−2002 and 2003−2012. Note that the maps do not show the regional abundance of the lichens as the density of sample plots is higher in southern regions than in northern regions (cf. Figure 1).


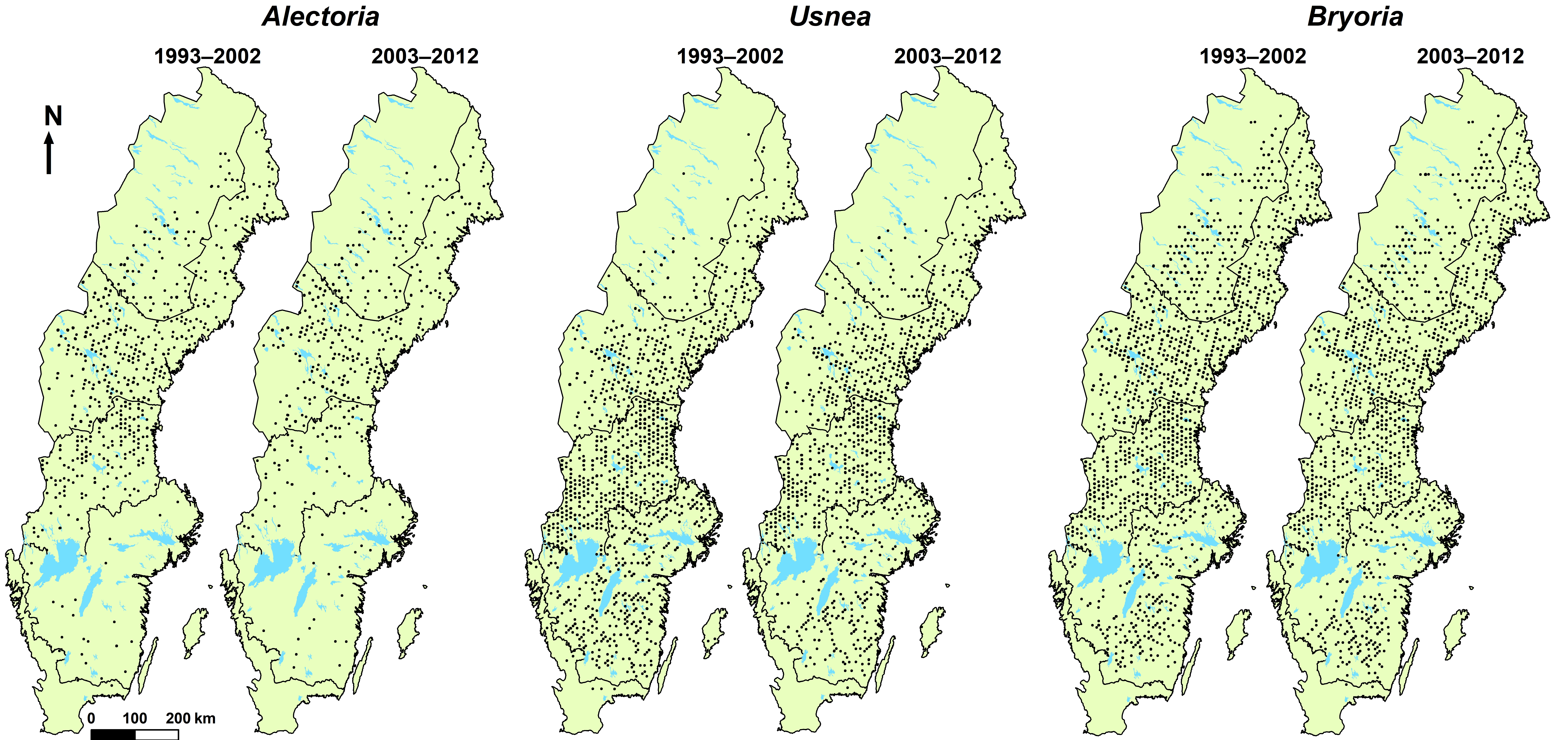


**Supporting Information Figure S2.** Estimated length (means and 95% CIs) of studied lichens on *Picea* in two 10-year time periods by five regions in Sweden. The estimates are based on trees with presence of the lichens. Stars indicate changes that are significant at *P* <0.05.


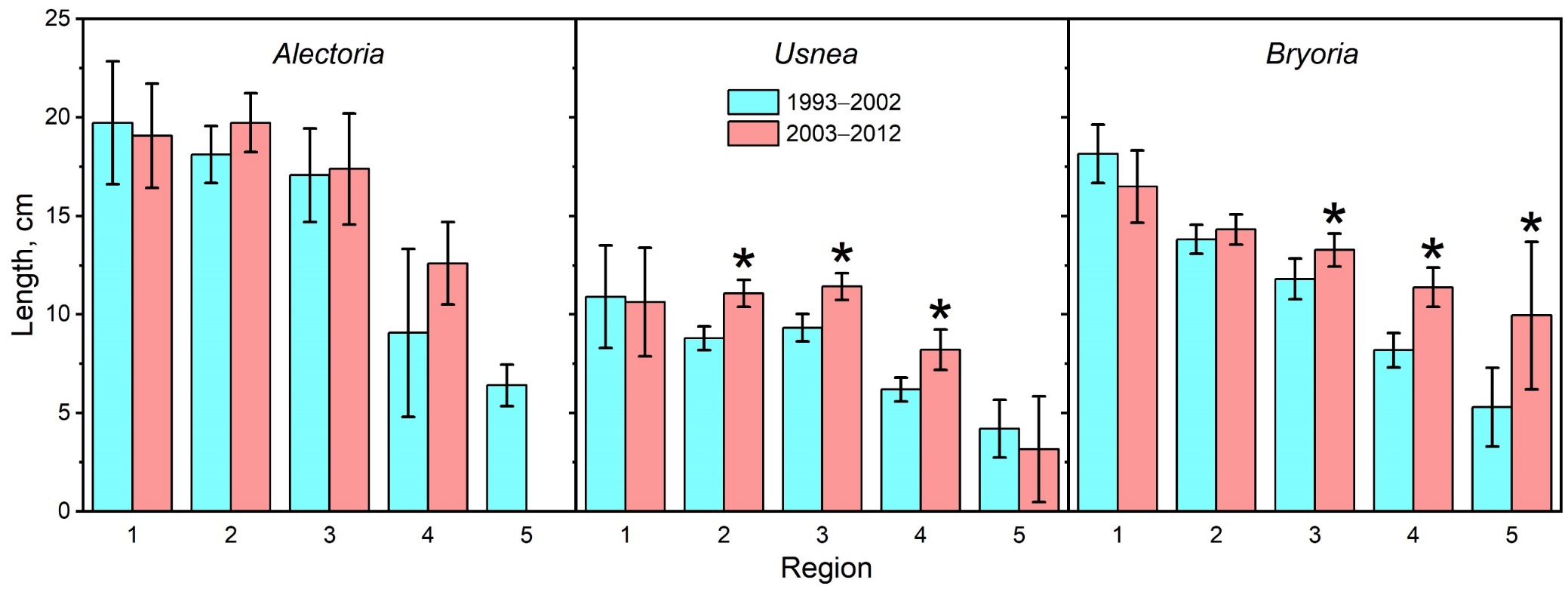

Supplement: Supplementary file 1 — Fig S1‐S2 [file GCB-28-3293-s001.docx]
