# Supplementary material for: Multiple drivers of large‐scale lichen decline in boreal forest canopies
Source: Glob Chang Biol. 2022 Mar 8;28(10):3293–309. doi: 10.1111/gcb.16128 (PMC9310866; doi:10.1111/gcb.16128)
Supplement: Supplementary file 2 — Table S1‐S5 [file GCB-28-3293-s002.docx]

**Supporting Information Table S1.** Number of sampled *Picea* of different categories in two time-periods (IP1, 1993−2002; IP2, 2003−2012) by region, and across all regions. Total *N* = 6140.

| Sample tree category | Region, no. of trees | |  |  |  |  |
| --- | --- | --- | --- | --- | --- | --- |
|  | 1 | 2 | 3 | 4 | 5 | 1−5 |
| 1. IP1 total (= 2 + 3 + 4 + 5) | 284 | 1053 | 883 | 1741 | 365 | 4326 |
| 2. Inventoried only in IP1 (% of 1) | 71 (25.0) | 254 (24.1) | 254 (28.8) | 517 (29.7) | 131 (35.9) | 1227 (28.4) |
| 3. Died after IP1 (% of 1) | 3 (1.1) | 29 (2.8) | 12 (1.4) | 28 (1.6) | 4 (1.1) | 76 (1.8) |
| 4. Cut after IP1 (% of 1) | 25 (8.8) | 131 (12.4) | 158 (17.9) | 366 (21.0) | 75 (20.5) | 755 (17.5) |
| 5. Remeasured in IP2 (% of 1) | 185 (65.1) | 639 (60.7) | 459 (52.0) | 830 (47.7) | 155 (42.5) | 2268 (52.4) |
| 6. New in IP2 (% of 7) | 88 (32.2) | 396 (38.3) | 351 (43.3) | 778 (48.4) | 201 (56.5) | 1814 (44.4) |
| 7. IP2 total (= 5 + 6) | 273 | 1035 | 810 | 1608 | 356 | 4082 |

**Supporting Information Table S2.** Estimated occurrence proportion (mean ±1SE) of studied lichens on *Picea* in the two periods (IP1, 1993−2002; IP2, 2003−2012) by region, and across all regions. Estimates for IP2 were divided into remeasured and new trees. Bold values for IP2 are significantly different (*P*<0.05) from the values for IP1.

| Lichen, period, tree category | Region |  |  |  |  |  |
| --- | --- | --- | --- | --- | --- | --- |
|  | 1 | 2 | 3 | 4 | 5 | 1−5 |
| *Alectoria* |  |  |  |  |  |  |
| IP1, all | 0.393±0.040 | 0.395±0.023 | 0.232±0.020 | 0.009±0.003 | 0.005±0.003 | 0.205±0.009 |
| IP2, all | 0.355±0.042 | 0.347±0.022 | **0.112**±0.017 | 0.014±0.003 | 0.000±0.000 | **0.173**±0.009 |
| IP2, remeasured | 0.411±0.051 | 0.389±0.028 | 0.121±0.021 | 0.026±0.007 | 0.000±0.000 | 0.219±0.013 |
| IP2, new | 0.269±0.064 | 0.301±0.031 | 0.104±0.022 | 0.006±0.002 | 0.000±0.000 | 0.130±0.011 |
|  |  |  |  |  |  |  |
| *Usnea* |  |  |  |  |  |  |
| IP1, all | 0.214±0.039 | 0.506±0.023 | 0.685±0.022 | 0.218±0.014 | 0.038±0.012 | 0.388±0.010 |
| IP2, all | 0.210±0.037 | 0.519±0.024 | **0.546**±0.026 | **0.152**±0.013 | **0.008**±0.004 | **0.342**±0.011 |
| IP2, remeasured | 0.199±0.040 | 0.556±0.028 | 0.660±0.030 | 0.186±0.018 | 0.019±0.011 | 0.400±0.013 |
| IP2, new | 0.226±0.061 | 0.479±0.034 | 0.440±0.035 | 0.128±0.017 | 0.001±0.001 | 0.289±0.014 |
|  |  |  |  |  |  |  |
| *Bryoria* |  |  |  |  |  |  |
| IP1, all | 0.983±0.009 | 0.854±0.016 | 0.619±0.024 | 0.168±0.013 | 0.005±0.003 | 0.532±0.008 |
| IP2, all | **0.849**±0.035 | **0.800**±0.018 | 0.565±0.026 | 0.165±0.013 | 0.015±0.010 | **0.506**±0.010 |
| IP2, remeasured | 0.876±0.037 | 0.811±0.021 | 0.639±0.030 | 0.191±0.020 | 0.000±0.000 | 0.575±0.012 |
| IP2, new | 0.807±0.058 | 0.788±0.027 | 0.496±0.036 | 0.146±0.016 | 0.024±0.017 | 0.443±0.013 |
|  |  |  |  |  |  |  |

**Supporting Information Table S3.** Correlation coefficients (*r*) between explanatory variables (in untransformed form) for remeasured trees, calculated without taking the sampling design into account. Bold values show correlations that are significantly different from zero at *P* <0.05. *N* = 2196).

| Variable | DBH1 | DBH∆ | CRL1 | CRL∆ | BAS1 | BAS∆ | AGE1 | AGE∆ | MAT | TEMP1 | TEMP∆ | RAIN1 | RAIN∆ | NDEP1 |
| --- | --- | --- | --- | --- | --- | --- | --- | --- | --- | --- | --- | --- | --- | --- |
| DBH∆ | **0.062** | − | − | − | − | − | − | − | − | − | − | − | − | − |
| CRL1 | **0.181** | **-0.187** | − | − | − | − | − | − | − | − | − | − | − | − |
| CRL∆ | **-0.049** | **0.170** | 0.022 | − | − | − | − | − | − | − | − | − | − | − |
| BAS1 | **0.182** | -0.019 | **0.488** | **0.164** | − | − | − | − | − | − | − | − | − | − |
| BAS∆ | **-0.052** | **0.121** | **-0.076** | 0.029 | **-0.381** | − | − | − | − | − | − | − | − | − |
| AGE1 | **0.141** | **-0.529** | **0.091** | **-0.258** | **-0.066** | **-0.123** | − | − | − | − | − | − | − | − |
| AGE∆ | -0.009 | **0.146** | -0.009 | **0.081** | -0.006 | 0.038 | -0.017 | − | − | − | − | − | − | − |
| MAT | **0.081** | **-0.309** | **0.132** | **-0.084** | 0.029 | **-0.108** | **0.359** | 0.022 | − | − | − | − | − | − |
| TEMP1 | **0.142** | **0.421** | **0.187** | **0.241** | **0.323** | 0.028 | **-0.537** | -0.006 | **-0.156** | − | − | − | − | − |
| TEMP∆ | **-0.089** | **-0.343** | **-0.160** | **-0.190** | **-0.259** | 0.027 | **0.447** | 0.018 | **0.122** | **-0.789** | − | − | − | − |
| RAIN1 | **0.130** | **0.297** | **0.135** | **0.212** | 0.215 | 0.024 | **-0.352** | -0.021 | **-0.091** | **0.696** | **-0.497** | − | − | − |
| RAIN∆ | 0.032 | **0.065** | -0.040 | 0.014 | -0.011 | -0.014 | **-0.058** | 0.012 | -0.022 | **0.120** | **-0.090** | **0.305** | **−** | **−** |
| NDEP1 | **0.140** | **0.381** | **0.151** | **0.246** | **0.251** | 0.011 | **-0.451** | -0.010 | **-0.138** | **0.839** | **-0.691** | **0.910** | **0.245** | **−** |
| NDEP∆ | **-0.088** | **-0.241** | **-0.078** | **-0.178** | **-0.173** | 0.007 | **0.255** | 0.032 | **0.087** | **-0.474** | **0.344** | **-0.834** | **-0.309** | **-0.805** |

**Supporting Information Table S4.** Pseudo *R*^2^, transformations and *P*-values for six explanatory variables in single variable multinomial logistic regression models predicting occurrence trajectories of studied lichens over a 10-year period on remeasured *Picea*. *P*-values for the regression coefficients are given for colonization, extinction, and persistence, in relation to absence (used as reference). *P* <0.05 are in bold. All other variables had *R*^2^ <0.05. *N* = 2196.

| Lichen, variable | Transformation | *P*-value |  |  |
| --- | --- | --- | --- | --- |
|  |  | Colonization | Extinction | Persistence |
| ***Alectoria*** |  |  |  |  |
| TEMP1 **(***R*^2^ = 0.155) | $x$ | 0.241 | **<0.001** | **0.006** |
|  | $x^{2}$ | **<0.001** | **<0.001** | **<0.001** |
| TEMP∆ **(***R*^2^ = 0.108) | $\left( 10x \right)^{-0.5}$ | **<0.001** | **<0.001** | **<0.001** |
| RAIN1 **(***R*^2^ = 0.092) | $x/1000$ | **<0.001** | **<0.001** | **<0.001** |
|  | $\left( x/1000 \right)^{2}$ | **<0.001** | **<0.001** | **<0.001** |
|  | $\left( x/1000 \right)^{3}$ | **<0.001** | **<0.001** | **<0.001** |
| NDEP1 **(***R*^2^ = 0.120) | $x/10$ | **<0.001** | **<0.001** | **<0.001** |
| NDEP∆ **(***R*^2^ = 0.055) | $x/10$ | 0.070 | **0.010** | **<0.001** |
|  | $\left( x/10 \right)^{2}$ | **0.001** | **0.002** | **<0.001** |
|  | $\left( x/10 \right)^{3}$ | 0.103 | 0.403 | **0.011** |
| AGE1 **(***R*^2^ = 0.077) | $x/100$ | **0.007** | **<0.001** | **<0.001** |
| ***Usnea*** |  |  |  |  |
| TEMP1 **(***R*^2^ = 0.127) | $x/10$ | **<0.001** | **<0.001** | **<0.001** |
|  | $\left( x/10 \right)^{2}$ | **<0.001** | **<0.001** | **<0.001** |
| TEMP∆ **(***R*^2^ = 0.020) | $\left( x \right)^{-1}$ | **<0.001** | **0.003** | **<0.001** |
| RAIN1 **(***R*^2^ = 0.065) | $\left( x/1000 \right)^{-1}$ | **<0.001** | **<0.001** | **<0.001** |
|  | $\left( x/1000 \right)^{-2}$ | **<0.001** | **<0.001** | **<0.001** |
| NDEP1 **(**0.126) | $\left( x/10 \right)^{-1}$ | **<0.001** | **<0.001** | **<0.001** |
|  | $\left( x/10 \right)^{-2}$ | **<0.001** | **<0.001** | **<0.001** |
| AGE1 **(***R*^2^ = 0.037) | $\left( x/100 \right)^{-0.5}$ | **0.001** | **<0.001** | **<0.001** |
|  | $x/100$ | **<0.001** | **<0.001** | **<0.001** |
| ***Bryoria*** |  |  |  |  |
| TEMP1 **(***R*^2^ = 0.278) | $x/10$ | 0.368 | 0.581 | 0.564 |
|  | $\left( x/10 \right)^{2}$ | **<0.001** | **<0.001** | **<0.001** |
| TEMP∆ **(***R*^2^ = 0.166) | $10x$ | **<0.001** | **<0.001** | **<0.001** |
| RAIN1 **(***R*^2^ = 0.185) | $\left( x/1000 \right)^{-1}$ | 0.922 | 0.388 | 0.986 |
|  | $\left( x/1000 \right)^{-2}$ | 0.299 | 0.512 | **0.009** |
| NDEP1 **(***R*^2^ = 0.270) | $\left( x/10 \right)^{-1}$ | **<0.001** | **<0.001** | **<0.001** |
| NDEP∆ **(***R*^2^ = 0.112) | $x/10$ | 0.133 | **0.026** | **0.049** |
|  | $\left( x/10 \right)^{2}$ | 0.052 | 0.062 | **0.015** |
|  | $\left( x/10 \right)^{3}$ | 0.103 | 0.403 | **0.011** |
| AGE1 **(***R*^2^ = 0.087) | $x/100$ | **0.007** | **<0.001** | **<0.001** |

**Supporting Information Table S5.** Pseudo *R*^2^ for final models and *P*-values for variable transformations included in multiple variable multinomial logistic regression models predicting occurrence trajectories of studied lichens over a 10-year period on remeasured *Picea*. *P*-values for the regression coefficients are given for colonization, extinction, and persistence, in relation to absence (used as reference). *P*-values <0.05 are in bold. *N* = 2196.

| Lichen, variable transformation | *P*-value |  |  |
| --- | --- | --- | --- |
|  | Colonization | Extinction | Persistence |
| ***Alectoria*** (*R*^2^ = 0.218) |  |  |  |
| Intercept | **0.001** | **0.048** | **0.001** |
| $(TEMP1)/10$ | 0.926 | **<0.001** | 0.131 |
| $\left( TEMP1/10 \right)^{2}$ | 0.235 | **<0.001** | **0.001** |
| $TEMP\Delta$ | 0.056 | 0.263 | 0.138 |
| $((TEMP\Delta)\times(AGE1)/100$) | **<0.001** | 0.132 | **0.036** |
| $(DBH\Delta)/100$ | 0.055 | **0.030** | **0.002** |
| $\left( RAIN1/1000 \right)^{-1}$ | **0.001** | **0.036** | **0.001** |
| $\left( RAIN1/1000 \right)^{-2}$ | **<0.001** | **0.031** | **0.001** |
| $(AGE1)/100$ | **0.012** | 0.178 | 0.357 |
| $(MAT)/100$ | **0.024** | 0.827 | **<0.001** |
|  |  |  |  |
| ***Usnea*** (*R*^2^ = 0.186) |  |  |  |
| Intercept | **<0.001** | **<0.001** | **<0.001** |
| $(TEMP1)/10$ | **<0.001** | **<0.001** | **<0.001** |
| $\left( TEMP1/10 \right)^{2}$ | **<0.001** | **<0.001** | **<0.001** |
| $\left( NDEP1/10 \right)^{-1}$ | **0.015** | **0.005** | **<0.001** |
| $\left( NDEP1/10 \right)^{-2}$ | **0.024** | **0.003** | **<0.001** |
| $DBH1$ | 0.130 | **0.014** | **<0.001** |
| $(DBH\Delta)/100$ | 0.194 | **<0.001** | **<0.001** |
| $(MAT)/100$ | **<0.001** | **0.003** | **<0.001** |
|  |  |  |  |
| ***Bryoria*** (*R*^2^ = 0.337) |  |  |  |
| Intercept | 0.070 | 0.161 | 0.803 |
| $(TEMP1)/10$ | 0.407 | 0.581 | 0.188 |
| $\left( TEMP1/10 \right)^{2}$ | 0.134 | 0.393 | 0.069 |
| $\left( TEMP1/10 \right)^{3}$ | **0.026** | 0.180 | **0.007** |
| $TEMP\Delta$ | 0.059 | 0.664 | **0.031** |
| $\left( NDEP1/10 \right)^{-1}$ | **<0.001** | **0.023** | **<0.001** |
| $(CRL1)/10$ | 0.757 | **0.016** | 0.594 |
| $\left( CRL1/10 \right)^{2}$ | 0.297 | **0.011** | 0.067 |
| $\left( AGE1/100 \right)^{-1}$ | 0.149 | **0.001** | **<0.001** |
| (MAT)/100 | **0.005** | 0.303 | **0.016** |
| (MAT)/($NDEP1$)/10 | **0.013** | 0.929 | 0.132 |
