# Supplementary material for: Multiple drivers of large‐scale lichen decline in boreal forest canopies
Source: Glob Chang Biol. 2022 Mar 8;28(10):3293–309. doi: 10.1111/gcb.16128 (PMC9310866; doi:10.1111/gcb.16128)
Supplement: Supplementary file 3 — Appendix S1 [file GCB-28-3293-s004.docx]

**Supporting Information Appendix 1. Computation of design weights for hair lichen trees in the Swedish National Forest Inventory.**

Here we provide details on how the design weights are computed for individual trees selected for measurement of hair lichens in the Swedish NFI. The total number of potential sample trees in the population is estimated by multiplying each selected tree’s observation with its design weight and then sum all the values. The occurrence proportion of each lichen is calculated as the ratio of the estimated number of trees with presence of the lichen to the estimated total number of potential sample trees in the population.

Let $A_{h}$ be the area of stratum $h$. The area of a tract in stratum $h$ is denoted by $a_{h}$ and the number of tracts in stratum $h$ is denoted by$k_{h}$. The sample selection consists of two phases. The subscript 1 denotes phase one and consist of the selection of the sample $s_{1}$ of $n_{1}$potential sample trees. Phase one consists of two stages, selection of tracts and then selection of potential sample trees. Let $q(i)$ denote the probability of selecting tree $i$ as a potential sample tree, given that tree $i$ has been selected in a tract. We also introduce the notation $h(i)$ as the stratum that tree $i$ belongs to and $t(i)$ as the tract that tree $i$ belongs to. The probability of including tree $i$ in $s_{1}$ is $\pi_{1i}=k_{h\left( i \right)}a_{h\left( i \right)}A_{h\left( i \right)}^{-1}q(i)$. Given $s_{1}$, a second-phase sample $s$ of size $n$ is drawn. Let $m_{t}$ be the number of trees selected for measuring lichen on tract $t$, and let $M_{t}$ denote the number of potential sample trees on tract $t$.

Phase two consists of selecting sample trees for measurement of lichen among the potential sample trees. Given that tree number $i\in s_{1}$, its conditional probability of inclusion in the second phase is $\pi_{i|s_{1}}=m_{t(i)}/M_{t(i)}$. Let $\pi_{i}^{*}=\pi_{1i}\pi_{i|s_{1}}$. Thus, the design weight for tree $i$ is ${1/\pi}_{i}^{*}$. See Särndal, Swensson & Wretman (2003, Ch. 9) for two-phase sampling and the $\pi^{*}$-estimator. The probabilities of including both tree$i$ and tree$j$ in the first and second phases are denoted by $\pi_{1ij}$ and $\pi_{ij|s_{1}}$ respectively. Let $\pi_{ij}^{*}=\pi_{1ij}\pi_{ij|s_{1}}$. There are four different cases for the joint inclusion probabilities of the trees$i$ and$j$.

Case 1: If $i$ and $j$ belong to different strata, then $\pi_{1ij}=\pi_{1i}\pi_{1j}$ and $\pi_{ij|s_{1}}=\pi_{i|s_{1}}\pi_{j|s_{1}}$.

Case 2: If $i$ and $j$ are the same tree, then $\pi_{1ii}=\pi_{1i}$ and $\pi_{ii|s_{1}}=\pi_{i|s_{1}}$.

Case 3: If $i\neq j$ and the trees belong to the same tract, then

$\pi_{1ij}=\frac{k_{h\left( i \right)}}{A_{h\left( i \right)}/a_{h\left( i \right)}}q(i)q(j)$ and $\pi_{ij|s_{1}}=\frac{m_{t(i)}}{M_{t(i)}}\frac{(m_{t\left( i \right)}-1)}{(M_{t\left( i \right)}-1)}$.

Case 4: If $i$ and $j$ belong to the same stratum but different tracts, then

$\pi_{1ij}=\frac{k_{h\left( i \right)}}{A_{h\left( i \right)}/a_{h\left( i \right)}}\frac{(k_{h\left( i \right)}-1)}{(A_{h\left( i \right)}/a_{h\left( i \right)}-1)}q(i)q(j)$ and $\pi_{ij|s_{1}}=\pi_{i|s_{1}}\pi_{j|s_{1}}$.

Assumptions of simple random sampling have been made for selection of tracts in the first phase and for selection of sample trees from the potential sample trees in the second phase. These assumptions affect only joint inclusion probabilities, used for variance estimation, and usually lead to conservative variance estimators.

The probability of selecting tree $i$ as a potential sample tree, $q\left( i \right)$, depends on the stratum $h(i)$ of tree $i$ and the diameter $d_{i}$ of tree $i$ (mm). Let $P1 = 1.0$ and $P4 = 2.3$ be constants. The constants $P10$ and $DM$ are different for different strata and are given in Table A1.

**Table A1.** The constants $P10$ and $DM$ for different strata.

| Stratum | $P10$ | $DM$ |
| --- | --- | --- |
| 1 | 2.90 | 410 |
| 2 | 2.75 | 435 |
| 3 | 2.60 | 460 |
| 4-5 | 2.50 | 490 |

For each strata, the constants $a1$, $b1$,$a10$, $b10$, are given by

$$a1=\frac{100\cdot P1-16\cdot P10}{99}$$

$$b1=\frac{16\cdot P10-P1}{9900}$$

$$a10=100-\frac{100\cdot P10}{{DM}^{2.5}-{100}^{2.5}}{DM}^{2.5}$$

$$b10=\frac{100-P10}{{DM}^{2.5}-{100}^{2.5}}$$

Finally, $q\left( i \right)$ is computed for different diameter classes according to the following. If $d_{i}<40$, then $q\left( i \right)=0$. If $40 \leq d_{i}<100$, then $q\left( i \right)=(a1+b1\cdot d_{i}^{2})/196$. If $100 \leq d_{i}\leq DM$, then $q\left( i \right)=(a10+b10\cdot d_{i}^{2.5})/100$. If $d_{i}\geq DM$, then $q\left( i \right)=1$.

**Reference**

Särndal, C. E., Swensson, B., & Wretman, J. (2003). *Model Assisted Survey Sampling*. Springer Science & Business Media.
