# Supplementary material for: Multiple drivers of large‐scale lichen decline in boreal forest canopies
Source: Glob Chang Biol. 2022 Mar 8;28(10):3293–309. doi: 10.1111/gcb.16128 (PMC9310866; doi:10.1111/gcb.16128)
Supplement: Supplementary file 4 — Appendix S2 [file GCB-28-3293-s003.pdf]

Supporting Information Appendix 2. Multinominal logistic regression under a two-phase sampling design

In a logistic regression model, a binary outcome variable Y is parameterized in terms of the logit of $Y = 1$ versus $Y = 0$. In a multi-outcome category model, where Y takes the values $Y = 0, ..., L-1$, we need $L-1$ logit functions (Hosmer et al. 2013). We use $Y = 0$ as the baseline outcome and form logit functions comparing each other category to it. Assume we have r covariates and a constant term, denoted by the vector $\boldsymbol{x}$, of length $r+1$, where $x_0 = 1$. The logit functions are then defined as

$$g_l(\boldsymbol{x}) = \log\left(\frac{P(Y = l|\boldsymbol{x})}{P(Y = 0|\boldsymbol{x})}\right) = \boldsymbol{x}'\boldsymbol{\beta}_l, \;\; l = 1, ..., L-1,$$

which gives the following conditional probability,

$$p_l(\boldsymbol{x}) = P(Y = l|\boldsymbol{x}) = e^{g_l(\boldsymbol{x})}\left(\sum_{m=0}^{L-1} e^{g_m(\boldsymbol{x})}\right)^{-1},$$

where $\boldsymbol{\beta}_0 = \boldsymbol{0}$ and $g_0(\boldsymbol{x}) = 0$.

The population-level log-likelihood function is

$$L(\boldsymbol{\beta}) = \sum_{i=1}^{N}\left\{\sum_{l=1}^{L-1} y_{il}g_l(\boldsymbol{x}_i) - \log\left(1 + \sum_{l=1}^{L-1} e^{g_l(\boldsymbol{x}_i)}\right)\right\},$$

where N denotes the population size, and the corresponding population-level estimating function system is

$$U_{lk}(\boldsymbol{\beta}) = \frac{\partial L(\boldsymbol{\beta})}{\partial \beta_{lk}} = \sum_{i=1}^{N} x_{ik}(y_{il} - p_l(\boldsymbol{x}_i)) = 0$$

for $l = 1, ..., L-1$, and $k = 0, ..., r$. The solution of this system is a finite population parameter $\boldsymbol{\beta}_N$, which is unknown to us.

Let π_i^* be defined as in Supporting Information Appendix 1. By using a sample s of trees selected for measuring hair lichen, where the trees are selected according to the sampling design used in the Swedish NFI, we obtain the pseudoscore

$$\hat{U}_{lk}(\boldsymbol{\beta}) = \sum_{i\in s} \frac{x_{ik}(y_{il} - p_l(\boldsymbol{x}_i))}{\pi_i^*}$$

for $l = 1, ..., L-1$, and $k = 0, ..., r$, which is an unbiased estimator of $\partial L(\boldsymbol{\beta})/\partial \beta_{lk}$ in the sense of Särndal et al. (1992; Result 9.3.1). An estimate of the finite population parameter $\boldsymbol{\beta}_N$ is obtained by setting the pseudoscores equal to 0 and solving for $\boldsymbol{\beta}$. We denote this estimator by $\hat{\boldsymbol{\beta}}_s$.

Let $\hat{\boldsymbol{U}}(\boldsymbol{\beta}) = [\hat{U}_{lk}(\boldsymbol{\beta})]$. The next step is to estimate the sampling variances and covariances of the parameter estimator. Binder (1983) suggested a solution to this problem using a multivariate version of Taylor series linearization. The result is a so-called sandwich-type variance estimator of the form

$$\widehat{\text{var}}(\hat{\boldsymbol{\beta}}_s) = \hat{\boldsymbol{J}}^{-1}\hat{\boldsymbol{V}}\hat{\boldsymbol{J}}^{-1},$$

where

$$\hat{\boldsymbol{J}} = \left.\frac{\partial \hat{\boldsymbol{U}}(\boldsymbol{\beta})}{\partial \boldsymbol{\beta}}\right|_{\boldsymbol{\beta}=\hat{\boldsymbol{\beta}}_s}$$

and $\hat{\boldsymbol{V}}$ is an estimator for the variance matrix of $\hat{\boldsymbol{U}}(\boldsymbol{\beta})$. For example, an unbiased estimator of the variance of $\hat{U}_{lk}(\boldsymbol{\beta})$ (in the sense of Särndal et al. (1992; Result 9.3.1 and Remark 9.3.1)) may be written as

$$\sum_{i \in s} \sum_{j \in s} \frac{\Delta^*_{ij}}{\pi^*_{ij}} \frac{x_{ik}(y_{il} - p_l(\boldsymbol{x}_i))}{\pi^*_i} \frac{x_{jk}(y_{jl} - p_l(\boldsymbol{x}_j))}{\pi^*_j},$$

where π^*_{ij} is defined as in Supporting Information Appendix 1 and $\Delta^*_{ij} = \pi^*_{ij} - \pi^*_i \pi^*_j$. Alternatively, in the spirit of Thompson (1997; Section 6.5.2), the variance of $\hat{U}_{lk}(\boldsymbol{\beta})$ may be estimated in a robust manner by

$$\sum_{i \in s} \left(\frac{x_{ik}(y_{il} - p_l(\boldsymbol{x}_i))}{\pi^*_i} \right)^2.$$

References

Binder, D. A. (1983). On the variances of asymptotically normal estimators from complex surveys. *International Statistical Review*, *51*, 279–292. https://doi.org/10.2307/1402588

Hosmer Jr, D. W., Lemeshow, S. A., & Sturdivant, R. X. (2013). *Applied Logistic Regression*, 3rd ed. Wiley, Hoboken.

Särndal, C. E., Swensson, B., & Wretman, J. (1992). *Model Assisted Survey Sampling.* Springer-Verlag, New York.

Thompson, M. E. (1997). *Theory of Sample Surveys.* Chapman & Hall, London.
